# Phylogenetic signatures reveal multilevel selection and fitness costs in SARS-CoV-2

**DOI:** 10.12688/wellcomeopenres.20704.1

**Published:** 2024-02-19

**Authors:** Vinicius Bonetti Franceschi, Erik Volz

**Affiliations:** 1 Department of Infectious Disease Epidemiology, School of Public Health, Imperial College London, London, England, W2 1PG, UK

**Keywords:** Molecular evolution, phylogenetic analysis, transmission fitness, natural selection, mutation, genetic clustering, within-host evolution, SARS-CoV-2

## Abstract

**Background:**

Large-scale sequencing of SARS-CoV-2 has enabled the study of viral evolution during the COVID-19 pandemic. Some viral mutations may be advantageous to viral replication within hosts but detrimental to transmission, thus carrying a transient fitness advantage. By affecting the number of descendants, persistence times and growth rates of associated clades, these mutations generate localised imbalance in phylogenies. Quantifying these features in closely-related clades with and without recurring mutations can elucidate the tradeoffs between within-host replication and between-host transmission.

**Methods:**

We implemented a novel phylogenetic clustering algorithm ( mlscluster, https://github.com/mrc-ide/mlscluster) to systematically explore time-scaled phylogenies for mutations under transient/multilevel selection. We applied this method for a SARS-CoV-2 time-calibrated phylogeny with >1.2 million sequences from England, and characterised these recurrent mutations that may influence transmission fitness across PANGO-lineages and genomic regions using Poisson regressions and summary statistics.

**Results:**

We found no major differences across two epidemic stages (before and after Omicron), PANGO-lineages, and genomic regions. However, spike, nucleocapsid, and ORF3a were proportionally more enriched for TFP-homoplasies than other proteins. We provide a catalog of SARS-CoV-2 sites under multilevel selection, which can guide experimental investigations within and beyond the spike protein.

**Conclusions:**

This study highlights the existence of important tradeoffs between within-host replication and between-host transmission shaping the fitness landscape of SARS-CoV-2.

## Plain Language Summary

Viral mutations can potentially carry a transient advantage, being simultaneously favourable for replication within hosts (e.g. by evading host immune responses) and deleterious to transmission (e.g. by having reduced cell binding). To identify such mutations, called transmission fitness polymorphisms (TFPs), we developed a clustering algorithm entitled mlscluster that computes lineage-level statistics based on the number of descendants, persistence times, and growth rates of lineages in comparison with co-circulating lineages, which usually are different than expected in the presence of such TFPs. We then applied it to a representative SARS-CoV-2 time-scaled tree with >1 million whole-genome sequences from England.

Our statistical analysis suggested approximately constant levels of transient selection across waves driven by very distinct variants. It also showed that genomic regions of known functional significance such as spike, nucleocapsid, and ORF3a were enriched for TFPs. This is the first study to characterise SARS-CoV-2 recurrent mutations with complex fitness tradeoffs, highlighting the existence of important tradeoffs in selection between intrahost replication and inter-host transmission. It also provides target mutations for laboratory-based investigations of their impacts and mechanisms of interaction with human cells.

## Introduction

It is generally held that, for most pathogens, the majority of polymorphic sites within a genome are selectively neutral or under weak selection ^1^. However, some mutations may confer a large transient increase in fitness, being advantageous to viral replication within hosts but detrimental to transmission. For example, HIV-1 is subject to multilevel selection, evolving considerably faster within individuals than at the epidemic level ^2, 3^, and virus which is more highly diverged from the population consensus is less likely to be transmitted ^4^.

Regarding SARS-CoV-2, the immense amount of genomic data collected during the COVID-19 pandemic has provided valuable insights about the competing forces influencing viral evolutionary dynamics ^5– 10^, but these data also presented novel challenges due to the scarcity of methods able to provide scalability using the power of big data streams while retaining fine-scale inferences (e. g. investigating fitness cost of individual mutations) ^5, 11– 13^. Provided scalable analytic pipelines can be developed, data from densely sampled epidemics can enable the identification of recurrent mutations in different branches of the phylogenetic tree, which potentially arise convergently as a consequence of virus response to adaptive selective pressures within hosts.

A particular challenge has been to infer population structure and phenotypic differences (reflected by phylogenetic asymmetries and imbalances) from observed pathogen genealogies ^14^. Even when clades are distantly related, they can present very similar distributions of coalescent times and branch lengths ^15^, as well as the proportion of descendants, persistence time, and growth rates when compared with closely-related clades. Most importantly, mutations influencing virus transmission fitness are expected to affect the distribution of offspring ^5^, consequently generating localised and quantifiable imbalance in time-scaled phylogenies. Therefore, the quantification and comparison of these parameters can indicate if similar evolutionary, demographic, or epidemiological processes are shaping viral evolution across different clades of a genealogy.

In molecular epidemiological studies, a set of particularly scalable approaches have been developed based on the calculation of phylogenetic clusters comprising two or more closely related samples. The frequency of phylogenetic clustering in a sample is sometimes considered a proxy for high transmission rate, especially in HIV datasets ^16– 18^, and can potentially indicate spread efficiency of a particular genotype (e.g. HIV drug resistance-associated mutations [DRAMs]). Intuitively and by extension, transmissibility and within-host evolution between variants can be considered a proxy for overall fitness ^19^. Recently, a genetic clustering analysis of HIV-1 identified variants containing specific DRAMs in antiretroviral therapy (ART)-naive transmission networks that reduce transmission fitness and suggested a negative correlation between lower frequencies of rare polymorphisms and fitness advantage ^18^.

Currently, similar clustering analyses have not yielded major insights into negatively-selected variants in SARS-CoV-2 despite the collection of unprecedented numbers of whole-genome sequences ^5^. Furthermore, there is considerable scope to improve on distance-based genetic clustering methods because such approaches will potentially have poor specificity for variants that negatively influence fitness. During the past few years, positive and negative selection in SARS-CoV-2 have mainly been investigated using methods that rely on synonymous rate variation across sites/branches ^20, 21^, and results from these approaches on SARS-CoV-2 comprehensive datasets are available for comparison ^22^. However, methodology to identify mutations that potentially have a transient fitness advantage is still lacking.

We developed a tree-based clustering algorithm, available as open-source R package mlscluster ( https://github.com/mrc-ide/mlscluster) ^23^, to identify potential transmission fitness polymorphisms (TFPs) by computing and comparing simple statistics from the offspring of recurring clade-defining mutations in a time-scaled phylogeny. This approach complements standard procedures based on synonymous rate variation across sites/branches by highlighting variants which likely have different and/or competing selective pressures within and between hosts. We demonstrate its applicability through the analysis of a representative >1.2 million SARS-CoV-2 genomic dataset from England, which indicated slightly higher TFP-homoplasy enrichment on B.1.1.7 and AY.4.* lineages and across genomic regions of known functional significance such as spike (S), nucleocapsid (N), ORF3a, ORF7, and ORF8. By providing a comprehensive catalog of the main sites driving multilevel selective pressures throughout the SARS-CoV-2 genome, we also expand the understanding of SARS-CoV-2 fitness landscape outside the well-studied spike protein. Therefore, these results can guide experimental studies on the functional impact of specific mutations, especially the subset that is advantageous to within-host replication but detrimental to transmission. 

## Methods

### Terminology and tree-based clustering statistics for detecting localised imbalance

**Figure 1.**
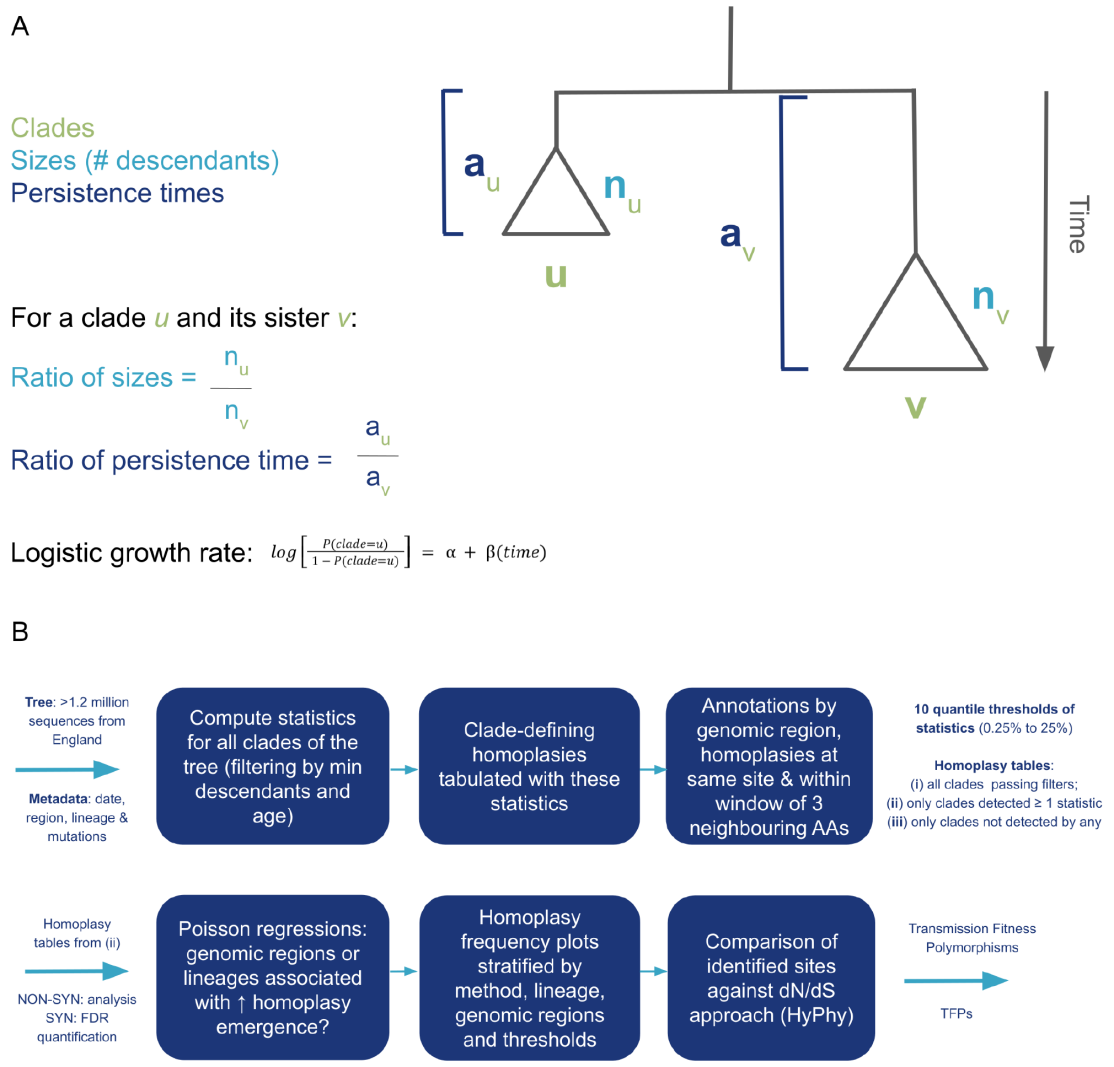
Schematic view of the tree-based clustering algorithm implementation and analytic pipeline. ( **A**) Main notations, parameters, and respective statistic formulas that are computed by mlscluster ( https://github.com/mrc-ide/mlscluster) for sister clades of the time-scaled phylogeny. ( **B**) Analysis workflow with main steps from input data to TFP inference.

We propose a phylogenetic tree-based clustering algorithm to systematically explore all nodes/clades in a time-scaled phylogenetic tree reconstructed from viral genomes. Assume two clades *u* (target clade) and *v* (comparator/sister) ( Figure 1A) organised in a time-scaled tree *t* and sharing ancestry ( *i.e.*, the same defining mutations). The size of each node ( *n* in Figure 1) is defined as the number of descendant sequences arising from it until the leaves of the tree are reached. The persistence time (given by *a* in Figure 1, is defined as the difference between the maximum sample time of samples descended from that node and the estimated time of the most recent common ancestor [tMRCA] of the node). After computing these simple parameters for each clade, the target nodes *u* are then contrasted against their comparators *v*, which can be their sister clade (the clade sharing an immediate ancestor assuming bifurcating phylogenetic relationship) or against all other clades sharing the same immediate ancestor (in case of polytomies/multifurcations).

If considering a node *u* with sister clade *v*, we compute three statistics based on these local phylogenetic patterns: (i) the ratio *S* of the number of samples descended from *u* and *v*, denoted *n _u_* and *n _v_*, which we will also call the ‘clade size’ ( Equation 1), (ii) the ratio *T* of persistence times denoted *a _u_* and *a _v_* ( Equation 2), and (iii) the logistic growth rate. The latter is defined as the coefficient of a logistic regression having a response variable defining sampling a descendent of *u* versus *v* and the sample time as a predictor ( Equation 3). The coefficient of such a logistic regression quantifies the relative growth of the clade and is related to the selection coefficient ^24^.


$$\;S_{uv}\;=\;\;\frac{n_{u}}{n_{v}}\;(1)$$

$$\;T_{uv}\;=\;\;\frac{a_{u}}{a_{v}}\;(2)$$

$$\log\;\left[\frac{P(\text{clade}\;=\;\text{u})}{1–P(\text{clade}\;=\;\text{u})}\right]\;\;=\;\;\alpha \;\;+\;\;\beta (\text{time})\;(3)$$

### Tree-based clustering algorithm implementation

The mlscluster method is implemented as an R package ( https://github.com/mrc-ide/mlscluster) ^23, 25^ that incorporates these multiple statistical methods for identifying especially convergently acquired mutations (homoplasies) that are detrimental for transmission (within a low quantile [e. g., 2%] of the probability distribution of at least one of the three statistics). These statistics applied to each clade are designed to be simple and computationally fast, making it possible to scan phylogenies with more than a million tips in hours using multiple CPU cores.

The clustering algorithm ( Figure 1B) starts by receiving a rooted bi- or multifurcating time-scaled tree ( *e. g.*, estimated using treedater ^26^, treetime ^27^ or chronumental ^28^) and associated metadata in a tabular format including sequence name, sample date, lineage, major lineage, and annotated mutations. The package then uses standard tree manipulation strategies implemented in the ape R package ^29^, particularly postorder traversal to visit nodes and tips based on the two-column edge matrix from the “phylo” class. Given this efficient way to visit nodes of the tree and edge lengths, we can easily extract the parameters of interest ( *e. g.*, time of the most recent common ancestor of each node, descendant identifiers and quantities, clade ages, etc). Target nodes are extracted based on the following conditions: (i) minimum number of descendants (ii) maximum number of descendants, (iii) minimum cluster age (in years), (iv) minimum sampling date, and (v) maximum sampling date. Only nodes passing all those criteria are kept for analysis.

Subsequently, target nodes and comparator (sister) clade(s) are gathered together and ratio of sizes, ratio of persistence time and logistic growth rates are calculated as previously stated. Since every sequence should include a metadata column (e.g. precomputed by COG-UK consortium, see Methods: Tree and metadata) listing mutations from its genome, the clustering algorithm tool incorporates a function to identify defining polymorphisms in target nodes. The mutation must be present in >75% of sequences in that node (while absent or in a smaller fraction than this percentage in its comparator) to be considered as defining, although this cutoff value can be changed. After computing clade-defining mutations, these are all tabulated and those which happen more than once in different nodes are retrieved as homoplasies. To enhance inspection of results, homoplasies are annotated into (i) regions of interest ( *e.g.* SARS-CoV-2 spike and nucleocapsid proteins), (ii) different mutations at the same site, and (iii) mutations within a 3 amino acid sliding window. There is also an additional sanity check for known sequencing artifacts ^30^ and for positively selected sites found by other analyses ^22^. Then, based on cut points dividing the range of the probability distribution of each statistic into continuous intervals with equal probabilities (quantiles), cluster thresholds can be specified to retain only clades potentially detrimental for transmission (default threshold of <1%) or carrying a positive fitness advantage (e.g., >99%). We intended to make the method flexible by creating a parameter that specifies in how many percentiles the statistic should be splitted ( *default* = 1 */*100) and another to keep values below or above the cutoff point.

Different comma-separated detailed outputs are generated for each of the three statistics showing nodes (and defining homoplasies) contained in the chosen cluster threshold, as well as the intersection (nodes identified by the threshold of the three statistics) and union (clades associated with at least one statistic threshold). Additionally, for each of the three homoplasy-annotated categories, three files are generated with their frequencies, and clades of occurrence considering (a) all target nodes that passed minimal filtering conditions, (b) only clades detected by one or more statistic threshold, (c) nodes not detected by any cutoff. Finally, these outputs are joined into one *data.frame* to facilitate further statistical analyses.

### Identifying potential TFPs using the mlscluster algorithm and a representative SARS-CoV-2 genomic dataset

***Tree and metadata.*** A global SARS-CoV-2 maximum likelihood (ML) phylogenetic tree and associated metadata including adm2 regions following the Database of Global Administrative Areas (GADM) subdivisions, PANGO-lineages, and annotated synonymous and non-synonymous mutations were obtained from the COVID-19 Genomics UK Consortium (COG-UK). From the ML tree, we estimated a time-scaled phylogeny using chronumental v0.0.53 ^28^. Only sequences from England were retained alongside the Wuhan/WH04/2020 (EPI_ISL_406801) reference sequence. In total, we included 1,275,669 sequences from all the 118 adm2 regions in England from June 01, 2020 to April 30, 2022, since they are associated with Pillar 2 representative community sampling efforts in the UK.

**Figure 2.**
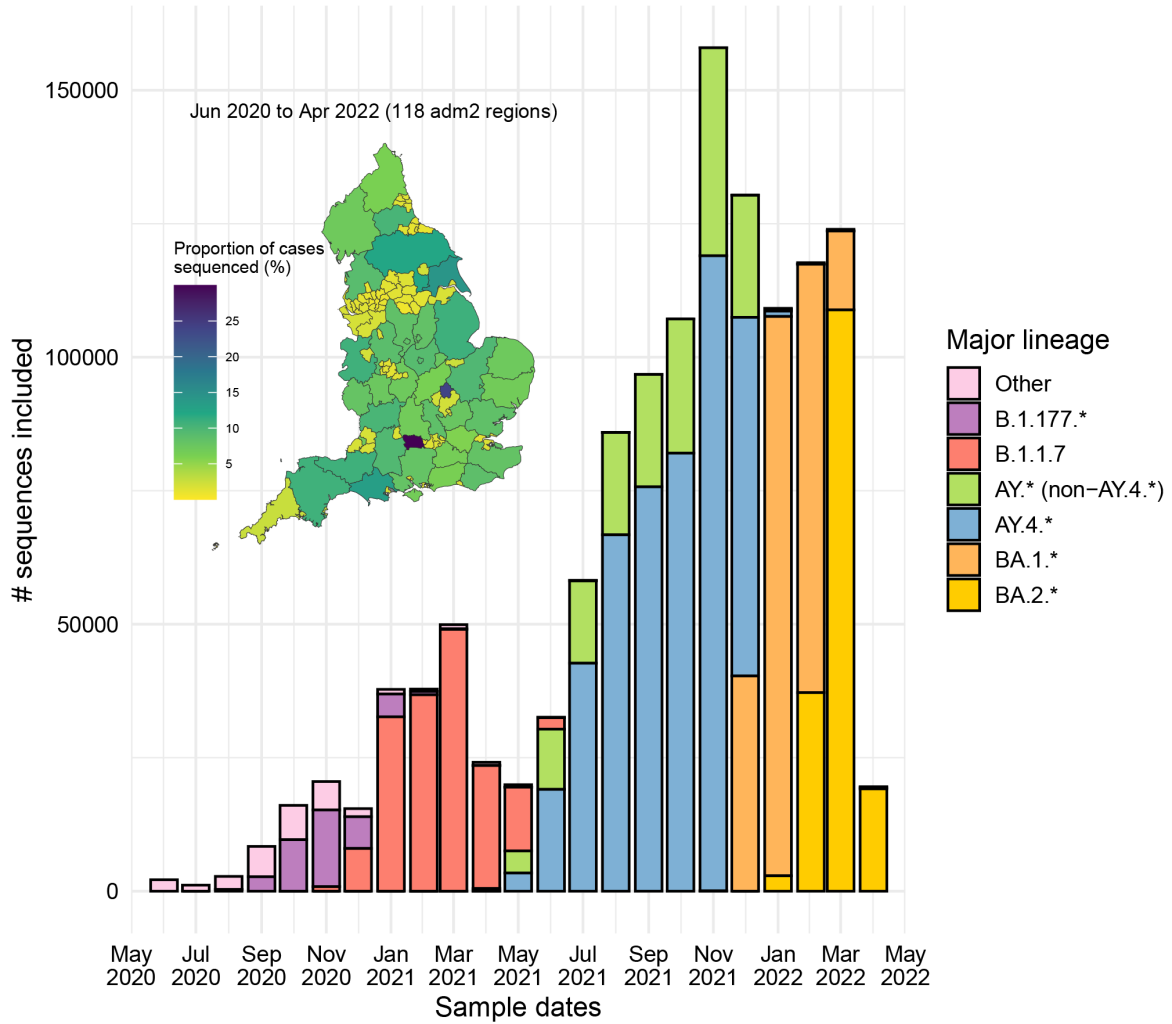
Spatiotemporal distribution of the SARS-CoV-2 sequences from England included in this study during the investigated period (June 2020 to April 2022). Main plot: Monthly-stratified frequency of the sequences stacked by major PANGO-lineage. Inset plot: Proportion of included sequenced cases across adm2 regions in England during the investigated period for 77% of the samples with unambiguous adm2-level assignments.

The proportion of cases sequenced for each region in England was computed by using UTLA and LTLA-level case counts obtained from the UK government website ( https://coronavirus.data.gov.uk/, accessed on 27 April 2023) matched against GADM adm2 geographical regions contained in COG-UK metadata. Since adm2, LTLA and UTLA regions are not entirely compatible, we have not considered on sequence counts samples with ambiguous matches (33%) for the map representation ( Figure 2).

### Statistical analysis for identifying genomic regions enriched for TFPs

We tested our approach using two COVID-19 pandemic time-periods: (i) from June 01, 2020 (including Wuhan/WH04/2020 reference sequence as root of the phylogeny) to November 15, 2022 (before Omicron BA.1.* variant emergence) (ii) from June 01, 2020 to April 30, 2022 (considering Omicron BA.1.* and up to Pillar 2 termination). For each period, 10 different thresholds (0.25, 0.5, 0.75, 1, 2, 3, 4, 5, 10, and 25%) of the clustering statistics are computed.

We also performed rigorous quality control to ensure our estimates were not biased by sequencing and base-calling artifacts. Firstly, we removed outlier sites highly enriched for homoplasies (above the 99% quantile of homoplasy frequencies for all thresholds), which we manually confirmed to be sequencing artifacts due to the high number of undetermined bases at respective sites in the alignment generating the phylogenetic tree. However, even after performing this approach, BA.1-defining mutations in the Receptor-binding Domain (RBD) were particularly identified as TFP-homoplasies for threshold=2% (Extended Data Figure S1A) ^31^, which was an unexpected result. To further inspect this inconsistency, we selected eight BA.1-spike defining mutations that were in our top100 of most frequent homoplasies (S:S371L, S373P, S375F, G496S, Q498R, N501Y, Y505H, N764K) and excluded all sequences (n=71,414, 5.6% of all sequences) that had undetermined bases (e.g. "NNN") in their respective codons from the nucleotide alignment. As a result, these sites were not detectable anymore (Extended Data Figure S1B) ^31^ and we could confirm they were the result of sequencing artifacts, which generally occur due to systematic differences in sequencing protocols and primer selection over time ^32^ and in different laboratories.

Consequently, we decided to perform a more aggressive quality control (henceforth called alignment-aware artifact removal) that has the advantage of not relying on excluding sequences without perfect coverage. First, we ran the mlscluster algorithm ( https://github.com/mrc-ide/mlscluster) without any artifact removal. We then extracted every homoplasic site detected and used seqtk v1.3-r106 ^33^ to create alignment files for each codon matching these sites in case of non-synonymous mutations and for each nucleotide in case of synonymous mutations. Afterwards, for each sequence, we added "X" (undetermined amino acid) for every site which had one or more non-ACTG character in respective codon positions, and "N" (undetermined nucleotide) for every non-ACTG synonymous site. Subsequently, we appended the "X" and "N" site annotations for all sequences within the existing metadata mutations column. We then incorporated a function named .fix_sites ( https://github.com/mrc-ide/mlscluster/blob/main/R/mlsclust.R#L314) to deal with those highly uncertain sites within the mlscluster package. In summary, since the first step to extract a defining mutation for each target clade is to compute the frequency of each mutation within the target and the comparator clades, we used the proportion of the most frequent mutation at a given site and added up the frequency of the "X" or "N", because it is most likely that the artifacts follow the majority. For example, if the target clade has the site S:G446 changing to S with frequency = 0.7 and to X with frequency = 0.3, we consider the S frequency = 1, and this mutation now has enough frequency (>75%) to be considered defining, which only would occur if the comparator has S:G446S at frequency <0.75%. In cases "X" and "N" are the most frequently mutated characters, the second-ranked amino acid or nucleotide at that site is added to these undetermined characters. For example, the target clade has the site S:N501 changing to X with frequency = 0.6 and to Y with frequency = 0.4, then the X frequency = 1. In such cases where either the target or comparator has a higher frequency of "X" or "N", the sites are not considered defining. A mutation is only considered defining when (i) it has one of the 20 valid amino acids (or stop codon) or the four valid nucleotides, (ii) it has a >75% frequency on the target clade and simultaneously <75% on the comparator node. This approach removed not only the eight previously investigated BA-1-spike defining mutations but also other six S sites for threshold = 2%, and affected mostly the BA.1 lineage without major changes for other lineages. Therefore, we consider that results arising from this alignment-aware artifact removal method are more reliable than previously and report those throughout the paper.

We performed Poisson regressions using the glm function from the stats package ^34^ and having the frequency of homoplasies as response variable ( Equation 4) to identify if any genomic regions and/or major PANGO-lineages were associated with increased TFP-homoplasy emergence for non-synonymous polymorphisms across different time periods and thresholds. A p-value ≤ 0.05 was considered statistically significant.


$$\begin{array}{l} \text{Freq}\_{\mathrm{homopl}}_{i\;}\sim \;\;\mathrm{Poisson}\;\left(\lambda_{i}\right)\\ \;\text{log}\;\left(\lambda_{i}\right)\;=\;\alpha_{j[i]}\\ \;+\;\beta_{1j[i]}(\text{major}\_\text{lineage})\\ \;+\;\beta_{2j[i]}(\text{genomic}\_\text{region})\\ \;+\beta_{3}(\text{indep}\_\text{positive}\_\text{selection}) \end{array}\;(4)$$

We assigned as major PANGO-lineages the following variants: B.1.177, Alpha (B.1.1.7), Delta AY.4 and sublineages (AY.4.*), other Delta (AY.* [non-AY.4.*]), Omicron BA.1.*, Omicron BA.2.*, and Others (all other lineages excluding recombinants). These were main drivers of epidemic waves in the UK and around the globe. Genomic regions included all 15 non-structural proteins (NSPs) from ORF1ab (NSP1-10, 12-16), S, ORF3a, Envelope (E), Membrane (M), ORF6, ORF7a, ORF7b, ORF8, N, and ORF10. Moreover, regions of characterised functional significance including the N-terminal Domain (NTD), the RBD and the Furin-cleavage site (FCS) of S, as well as the Linker Domain of N ^35^ were considered as additional genomic regions. The other covariate was whether the sites was independently found under selection based on a HyPhy-based synonymous rate variation across sites/branches analysis ^22^.

To further investigate the genomic regions enriched for TFP-homoplasies resulting from the Poisson regressions and to compare the sites identified as potentially under selection against results from the literature ^22^, we generated different exploratory visualisations using ggplot2 ^36^. These were stratified by the method of detection ( mlscluster, HyPhy, or both), major PANGO-lineages, genomic regions (including frequency normalisation by size), and cluster thresholds.

### Codon-aware false discovery rate (FDR)

We used synonymous homoplasies for characterising the FDR of our approach under the assumption that synonymous sites would not provide a fitness advantage. Since these sites represent one-third of the genome and mutations tend to occur in the third codon position to preserve the encoded amino acid, this weighting needs to be taken into account when computing FDR. Firstly, we defined the percentage of erroneous TFP calls for each threshold *t* as:


$$\;{\mathrm{FDR}}_{t}\;=\;\;\frac{Y_{t}}{i}\;\;\times \;\;100\;(5)$$

where *Y* is the number of TFP calls specifically among the *i* polymorphic third codon position sites with > 100 mutated sequences at the given site (considering the analysed > 1.2 million genomes). This is also performed for each SARS-CoV-2 protein. Multiplication by 100 transforms the probability of erroneously calling a TFP into a percentage for easier interpretation.

Similarly, a separate error rate ( *ε* or codon-aware FDR) is also computed relative to the sites at first and second codon positions as follows:


$$\;\varepsilon_{t}\;=\;\;\frac{{\mathrm{FDR}}_{t}\times \;\;Z_{t}}{j}\;\;(6)$$

where *Z* is the number of TFP calls at codon positions one and two, and *j* is the total number of polymorphic sites at first and second positions with > 100 mutated sequences at the given site.

Both calculations were performed for the two analysed time periods, with slightly smaller error rates for the timeframe before Omicron emergence.

## Results

We utilised our new approach ( Figure 1) ^31^ to analyse 1,275,669 SARS-CoV-2 whole genome sequences from England sampled between June 2020 and April 2022. This time period encompasses: (i) a period from June to December 2020 dominated by A.*, B.* and B.1.177 lineages, (ii) a timeframe between January and May 2021 when Alpha (B.1.1.7) predominated, (iii) a wave from June to December 2021 characterised by rapid spread of Delta (AY.4.* and other AY.*), and (iv) the Omicron (BA.1 and BA.2) epidemic cycle from December 2021 to April 2022 ( Figure 2). For clarity and due to the main patterns observed, the analytic period presented includes: (i) June 2020 to mid-November 2021 (pre-Omicron interval) and (ii) mid-November 2021 to April 2022 (including Omicron). Only data collected through community sampling (Pillar 2) were included to reduce bias towards more severe infections and avoid the inclusion of data that was collected for special purposes. The geographical representation of the data is similar across different regions of England, with a mean proportion of community cases having a sequence of 6.7% and median of 7.1% ( Figure 2, inset plot).

### Lineages and genomic regions enriched with SARS-CoV-2 TFP-homoplasies

**Figure 3.**
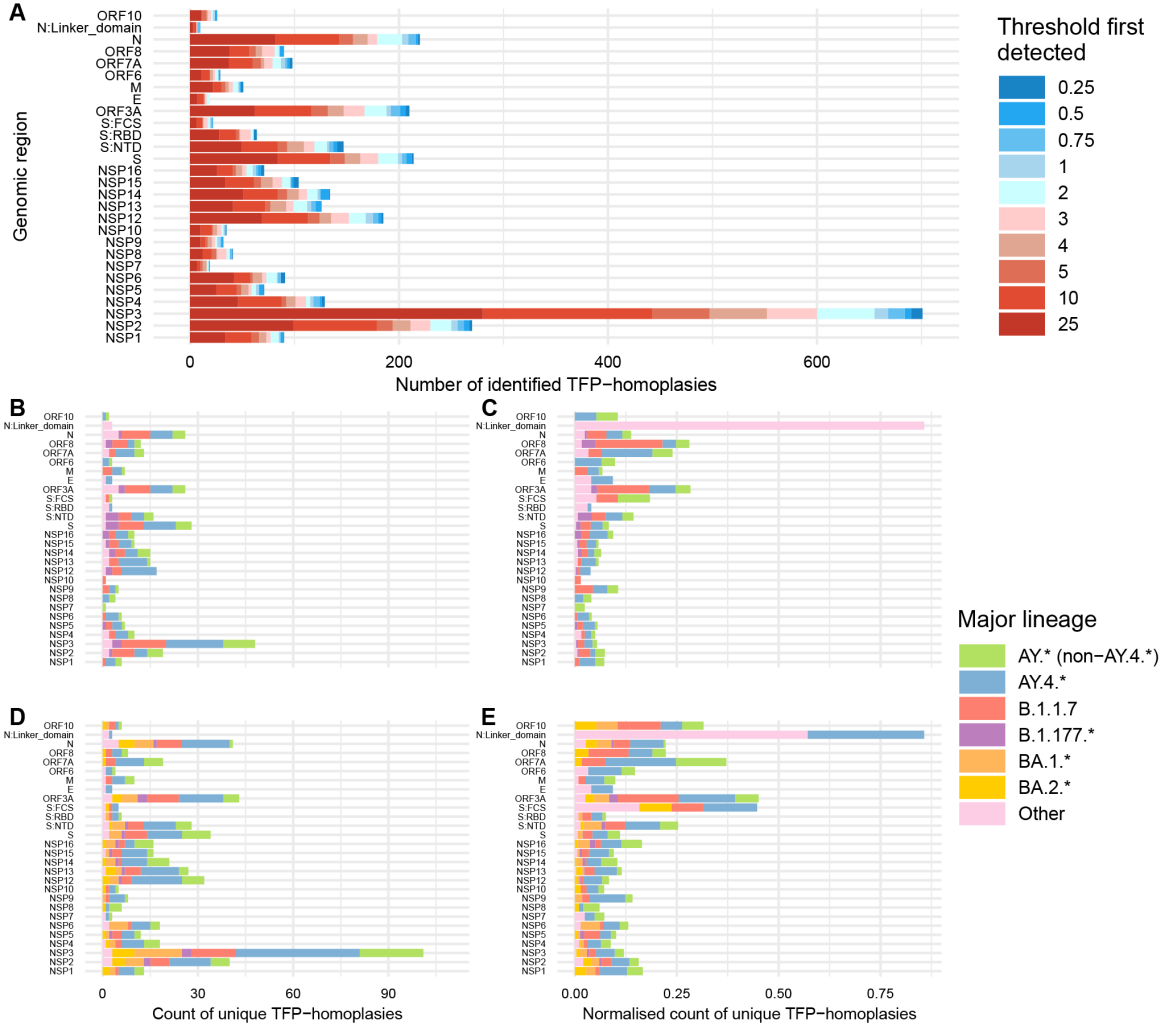
Frequency of SARS-CoV-2 TFP-homoplasies per genomic region considering all cluster thresholds and the more reliable threshold of 2%. ( **A**) Count of TFP-homoplasic sites for all SARS-CoV-2 proteins across the 10 different cluster thresholds ranging from the more (0.25%) to the less stringent (25%). ( **B**– **E**) Count of TFP-homoplasies per genomic region for two different time periods and considering threshold = 2%. ( **B**) Non-normalised counts per lineage for timeframe pre-Omicron (June 2020 to mid-November 2021). ( **C**) Normalised counts per lineage (divided by genomic size) for the same period as ( **B**). ( **D**) Non-normalised counts for the timeframe including Omicron (June 2020 to end of April 2022). ( **E**) Normalised counts for the same period as ( **D**).

The presence of recurring synonymous polymorphisms classified as TFP-homoplasies allowed us to investigate the FDR for each genomic region as a function of the applied cluster thresholds. The frequency of synonymous mutations along the genome (Extended Data Figure S2) ^31^ and the FDR across genomic regions (Extended Data Figure S3) ^31^ support that sites only detected at thresholds ≥ 10% must be investigated with caution since they generally have associated FDRs ≈ 40% and *ε* ≈ 15%, whereas cutoffs ≤ 2% retain an acceptable FDRs ≈ 10% and *ε* ≈ 2%. Additionally, these more erroneous thresholds (≥ 10%) represent > 50% of the identified TFP sites ( Figure 3A).

Therefore, among the ten cluster thresholds ranging from the more strict (0.25%) to the more lenient (25%) values (Extended Data Text S1, Extended Data Figure S4) ^31^, we report results with the 2% threshold and after performing rigorous quality control using an alignment-aware artifact removal method to represent sites under putative multilevel selection (see Methods: Statistical analysis for identifying genomic regions enriched for TFPs. With this threshold, the false discovery rate (FDR) and codon-aware FDR ( *ε*) (see Methods: Codon-aware false discovery rate (FDR)) are respectively around 10% and 2.5% (Extended Data Figure S3) ^31^. Results for sites identified with other thresholds are presented in the Extended Data ^31^.

For the period predating Omicron BA.1.* emergence, we found that B.1.1.7 was consistently the lineage with the highest coefficient for enrichment of TFP-homoplasies, reaching statistical significance for ≥ 2% thresholds. However, TFP enrichment was similar across lineages and not found for specific genomic regions (Extended Data File S1) ^31^. Although not significantly different, TFP-homoplasies were slightly more abundant in the small linker domain ^35^ of the N protein, ORF3a, and ORF8 for this time period ( Figure 3B and C).

All considered major lineages were associated with increased TFP-homoplasy emergence for ≥ 1% thresholds during the timeframe which includes Omicron BA.1.* as the dominant variant. Although not consistent for different thresholds, the rank of lineage coefficients was Other lineages > B.1.1.7 > AY.4.* > BA.1.* > AY.* (non-AY.4.*) for threshold = 2%. Once again, there were no statistically significant results at the 2% threshold regarding genomic regions (Extended Data File S2) ^31^. However, N:linker domain, ORF3a, S:FCS, ORF7a, and ORF10 presented a higher number of TPFs per site ( Figure 3D and E), which is relatively consistent with the preceding period.

Normalising homoplasy counts by the size of each genomic region (giving less weight to larger genomic regions) has a large influence in interpreting the relative rates of TFP acquisition. This is especially demonstrated by NSP3, which accrues more TFPs due to its size of 5835 nucleotides ( Figure 3B and D), but when normalised has a similar distribution of TFPs compared to other genomic regions ( Figure 3C and E).

### TFPs along the SARS-CoV-2 genome and comparison with other approaches for detecting sites under selection

**Figure 4.**
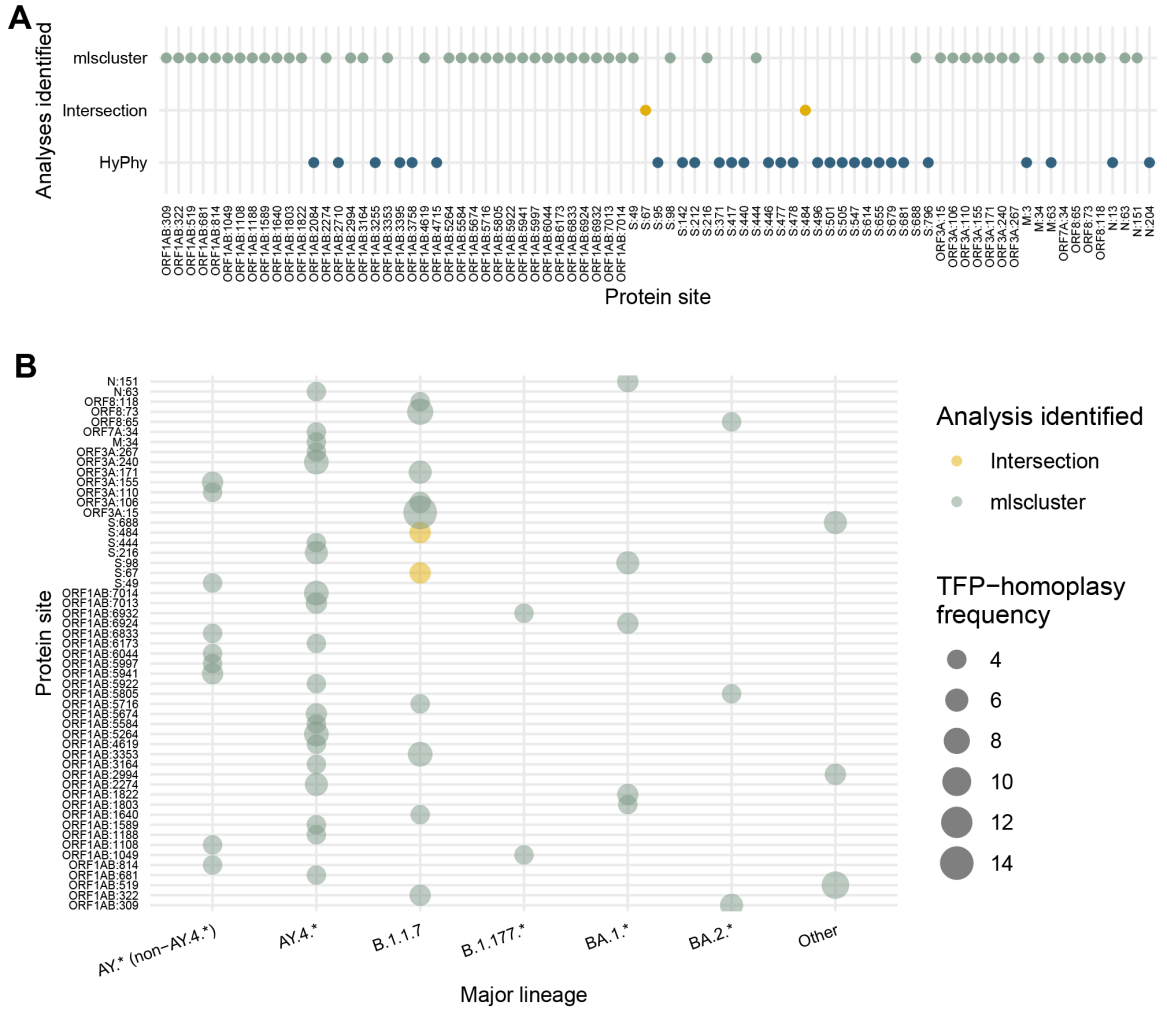
TFP-homoplasy identification compared to sites identified as under positive selection. Sites are compared across different major lineages. ( **A**) Comparison of top 30 identified sites under multilevel selection by our tree-based clustering approach for the whole-period (including Omicron) for cluster threshold = 2% against the HyPhy analysis ^22^, also presenting concordant results (intersection) between both methods. ( **B**) Bubble plot of TFP-homoplasy frequencies attributed to different major PANGO-lineages.

When comparing the 30 most frequent mlscluster TFP-homoplasies against the 30 mutations under positive selection detected by the HyPhy-based approach ^22^ for the cluster threshold = 2% and period before Omicron emergence, we detected three concordant sites (S:67, S:95, and S:484), 27 positively selected sites only detected by HyPhy, and 63 sites only detected by mlscluster statistics (Extended Data Figure S5) ^31^. For the timeframe including Omicron, we identified two sites under selection concordantly between methods and with the previous period (S:67 and S:484), 28 discordant results, and 51 new potential TFPs across seven proteins/ORFs ( Figure 4A).

The top 30 most frequent TFP-homoplasies across lineages shows that the B.1.1.7 (n=22) and the AY.4.* (n=21) were similarly enriched for those highly-frequent TFP-homoplasies up to mid-November 2021 (Extended Data Figure S5) ^31^, while AY.4.* (n=20) was notably the major lineage harbouring more TFPs ( Figure 4B, Table 1) when considering Omicron BA.1.*. Morever, the analysis of lineage-specific top 30 TFP-homoplasies regardless of threshold shows that less than half of those are firstly detected on smaller cluster thresholds (up to 2%) (Extended Data Figures S6-S10) ^31^.

**Table 1.** Top 30 TFP-homoplasies within the spike protein for the period between June 2020 and April 2022 (including Omicron) and cluster threshold = 2%.

Homoplasy	Frequency	Major lineage	HyPhy	Genomic region	Amino acid length of protein
ORF3A:L15F	14	B.1.1.7	No	ORF3A	275
ORF1AB:G519S	9	Other	No	NSP2	638
ORF8:Y73C	8	B.1.1.7	No	ORF8	121
ORF1AB:H5264Y	7	AY.4.*	No	NSP12	932
ORF1AB:K3353R	7	B.1.1.7	No	NSP5	306
ORF1AB:R7014N	7	AY.4.*	No	NSP16	298
ORF3A:P240S	7	AY.4.*	No	ORF3A	275
ORF1AB:P309L	6	BA.2.*	No	NSP2	638
ORF1AB:T2274I	6	AY.4.*	No	NSP3	1945
ORF3A:S171L	6	B.1.1.7	No	ORF3A	275
**S:A688V**	6	Other	No	S:FCS	38
**S:L216F**	6	AY.4.*	No	S:NTD	292
**S:S98F**	6	BA.1.*	No	S:NTD	292
N:P151L	5	BA.1.*	No	N	413
ORF1AB:A2994V	5	Other	No	NSP4	500
ORF1AB:K322R	5	B.1.1.7	No	NSP2	638
ORF1AB:L6924F	5	BA.1.*	No	NSP16	298
ORF1AB:P7013L	5	AY.4.*	No	NSP16	298
ORF1AB:S5674L	5	AY.4.*	No	NSP13	601
ORF1AB:T1822I	5	BA.1.*	No	NSP3	1945
ORF1AB:T5941I	5	AY.* (non-AY.4.*)	No	NSP14	527
ORF3A:D155Y	5	AY.* (non-AY.4.*)	No	ORF3A	275
ORF3A:L106F	5	B.1.1.7	No	ORF3A	275
**S:A67V**	5	B.1.1.7	Yes	S:NTD	292
**S:E484K**	5	B.1.1.7	Yes	S:RBD	223
M:L34F	4	AY.4.*	No	M	222
N:D63G	4	AY.4.*	No	N	413
ORF1AB:A1049V	4	B.1.177.*	No	NSP3	1945
ORF1AB:A5922V	4	AY.4.*	No	NSP13	601
ORF1AB:A6044V	4	AY.* (non-AY.4.*)	No	NSP14	527
ORF1AB:D5584Y	4	AY.4.*	No	NSP13	601
ORF1AB:G6173V	4	AY.4.*	No	NSP14	527
ORF1AB:H1108Y	4	AY.* (non-AY.4.*)	No	NSP3	1945
ORF1AB:L681F	4	AY.4.*	No	NSP2	638
ORF1AB:M5997I	4	AY.* (non-AY.4.*)	No	NSP14	527
ORF1AB:P1640S	4	B.1.1.7	No	NSP3	1945
ORF1AB:P1803S	4	BA.1.*	No	NSP3	1945
ORF1AB:P4619L	4	AY.4.*	No	NSP12	932
ORF1AB:P6932S	4	B.1.177.*	No	NSP16	298
ORF1AB:R3164H	4	AY.4.*	No	NSP4	500
ORF1AB:R5716C	4	B.1.1.7	No	NSP13	601
ORF1AB:S1188L	4	AY.4.*	No	NSP3	1945
ORF1AB:T1589I	4	AY.4.*	No	NSP3	1945
ORF1AB:T5805M	4	BA.2.*	No	NSP13	601
ORF1AB:T6833I	4	AY.* (non-AY.4.*)	No	NSP16	298
ORF1AB:T814I	4	AY.* (non-AY.4.*)	No	NSP2	638
ORF3A:A110S	4	AY.* (non-AY.4.*)	No	ORF3A	275
ORF3A:P267L	4	AY.4.*	No	ORF3A	275
ORF7A:P34L	4	AY.4.*	No	ORF7A	121
ORF8:A65V	4	BA.2.*	No	ORF8	121
ORF8:L118V	4	B.1.1.7	No	ORF8	121
**S:H49Y**	4	AY.* (non-AY.4.*)	No	S:NTD	292
**S:K444R**	4	AY.4.*	No	S:RBD	223

When expanding to the top 100 TFP-homoplasies (Extended Data Table S1) ^31^, 21 sites are located within the larger NSP3 which has 1945 amino acids, 14 are from ORF3a (275 sites), 12 from spike excluding NTD, RBD, and FCS (721 amino acids), nine from NTD (292 sites), four from FCS (38 amino acids), two from RBD (223 sites), and seven from the N protein (420 amino acids). Respectively, AY.4.* and AY.* account for 50 and 28 of these top 100 sites, followed by B.1.1.7 (n=24).

A manual inspection of TFP-homoplasies with frequency ≥ 5 (Extended Data Table S1) ^31^ confirmed that they emerged independently in multiple lineages during the pandemic and are predominantly found in extremely low (<1%) frequencies. This independent analysis provides additional evidence that their evolution is consistent with a transient selective pressure. Additionally, it shows that the impact of very few mutations outside the S protein have been characterised experimentally (Extended Data Table S1) ^31^.

**Figure 5.**
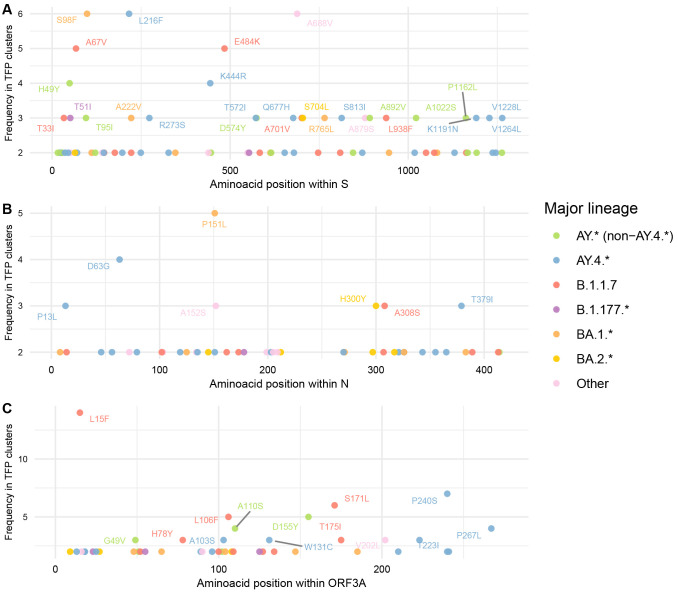
Frequency of identified TFP-homoplasies alongside genomic regions with major functional significance and normalised counts for cluster threshold = 2% and period including Omicron. ( **A**) Spike. ( **B**) Nucleocapsid. ( **C**) ORF3a. TFPs are coloured by major PANGO-lineage and annotated if frequency > 2.

By focusing on individual proteins that harbour a major functional significance and higher normalised count of TFPs (S, N, ORF3a, ORF7a, and ORF8), we highlight relevant sites under multilevel selection for further experimental investigations. These sites include S:A67V, S:S98F, S:L216F, S:E484K, S:A688V, N:P151L, ORF3a:L15F, ORF8:Y73C, etc. Additionally, these transient selective processes are more likely to be acting uniformly across each protein and not in specific hotspots ( Figure 5, Extended Data Figure S11, Extended Data Table S1) ^31^.

## Discussion

We have quantified transient selective forces acting on SARS-CoV-2 lineages and mutations through the calculation of three statistics (ratio of sizes, ratio of persistence time and logistic growth rates between sister clades) and extraction of clades containing values of those statistics below small cluster threshold cutoffs. To mitigate the inclusion of spurious sites, we included only recurring clade-defining mutations (homoplasies) across cluster thresholds with low associated FDRs and excluded probable sequencing artifacts. To the best of our knowledge, this is the first attempt to identify SARS-CoV-2 polymorphisms that negatively influence transmission fitness while being beneficial for within-host replication. Our tree-based clustering approach provides a scalable way to analyse huge genomic datasets with >1 million sequences for multilevel selection while also accounting for shared ancestry.

Although the COVID-19 pandemic offered the opportunity to collate genomic datasets of unprecedented sizes, estimating the transmission fitness of individual polymorphisms in this context is challenging. In the early epidemic, inference of sites under positive selection was hampered by low sensitivity given the small genetic diversity of the virus. For example, a phylogenetic approach was developed to quantify imbalance in clades containing recurrent mutations ^5^, and this approach found a lack of evidence for increased transmissibility from recurrent SARS-CoV-2 mutations. However, this approach only used ≈50,000 sequences up to July 2020 and has not considered persistence times and growth rates as measures of differential fitness across clades of the phylogeny. After the emergence of VOCs with elevated substitution rates, other attributes such as convergent evolution, sparse sampling, and vaccine-elicited immunity appeared as relevant confounding factors. Most importantly, the detection of positive selection does not necessarily imply enhanced transmissibility, and the effects of individual mutations on this trait will typically be modest ^6^.

Genetic diversity in an infected individual is governed by repeated cycles of within-host (e.g. replication and immune escape pressures) and between-host processes (e.g. transmission bottlenecks), with the outcome of selection at each level having an effect on the other ^37^. The rapid accumulation of mutations in individuals ^38, 39^ with long-lasting chronic SARS-CoV-2 infection is hypothesised to contribute to the emergence of variants such as Alpha and Omicron ^40^. Thus the interaction of within-host and between-host selective processes can occasionally have very large epidemic-level effects.

The inspection of the global and lineage distributions of highly frequent TFP-homoplasies confirmed that these mutations generally emerge independently in multiple lineages but remain quite rare, which is consistent with a simultaneous within-host advantage and between-host disadvantage. This systematic investigation also emphasises the scarcity of experimental studies to characterise the functional impact of mutations outside the spike protein.

Our approach identified modest differences in multilevel selection signals across two different epidemic phases, lineages and genomic regions in the UK. We hypothesised that transient selective forces would become stronger after high-levels of convalescent and vaccine-induced immunity have been reached, but our results do not support this hypothesis. Our observation of approximately constant levels of transient selection across waves driven by extremely distinct variants may in part be driven by long-duration chronic infections which occur at low frequency and provide greater opportunities for accelerated within-host evolution favouring immune evasion. Our data did not include clinical covariates that would allow us to investigate the association of chronic infection or duration of infection and the presence of TFPs.

Sequencing of chronically-infected patients throughout multiple time points of their long-lasting infection provided external validation of our observed patterns. Nucleotide substitution rates were around twice as fast during chronic infections when compared with the global SARS-CoV-2 evolutionary rate ^41^. Additionally, mutations identified in the top 100 most frequent TFP-homoplasies by our approach such as S:E484K ^41– 44^, S:T95I ^43, 44^, ORF8:Y73C ^42^, ORF8:L118V

^41^, ORF1ab:S944L ^41^, and ORF1ab:T1543I ^41^ also

emerged after days of chronic infection. Although usually associated with immune escape and increased ACE2 affinity, these recurrent mutations lack the capacity to enhance transmission ^43, 44^ as demonstrated by their low epidemic-level frequency after multiple independent occurrences. Additionally, distinct viral populations appear to be residing in different niches ( *e.g.* organs) of a patient’s body ^44^ and an impaired immune system selects for mutations that confer intra-host replication and persistence ( *e.g.* immune evasion) as opposed to general acute infections, in which mutations favouring inter-host transmission are a major target of selective pressures ^43^.

Despite distance-based clustering in HIV networks having been extensively used as a proxy for transmissibility, this approach is generally based on a cutoff from pairwise distances separating sequences ^16^. Consequently, poor specificity for variants negatively influencing fitness is evident (i) when a variant is isolated, occurring along a long branch not captured by distance threshold, (ii) when a variant is imported, and large genetic distances can reflect unsampled diversity in the country of origin or a rare recombination or hypermutation event, not necessarily reflecting a fitness cost. Our method addresses these limitations by incorporating the number of descendants, persistence times, and growth rates across sister clades with and without the mutations under investigation, and using independently-acquired substitutions to remove spurious relationships. Introducing these multiple sources of information can provide more accurate estimates, but also introduce biases. Primarily, our analysis is sensitive to sequencing artifacts. Although we used data from a highly standarised sequencing consortium (COG-UK), changes in primer sets after Omicron emergence ^32^, as well as sequencing coverage and base-calling errors can potentially influence our conclusions, as demonstrated by our several quality control and artifact removal methods employed. A second caveat arises from the assumption of representative sampling. Although we utilised data from England during a period of proportional (to cases) community sampling to minimise this effect, the rate of sampling varied substantially over time and further analyses are needed to investigate the impact of non-representative sequencing in our approach.

## Conclusions

We developed a method capable of identifying sites under multilevel selection from >1.2 million SARS-CoV-2 sequences using rigorous quality control, statistical tests, and control for false detection. The comprehensive catalog of TFPs identified here and especially abundant in S, N, ORF3a, ORF7, and ORF8 highlight the existence of important tradeoffs between within-host replication and between-host transmission of SARS-CoV-2 that may warrant further experimental investigation.

## Data Availability

Zenodo: Underlying and Extended data for: Phylogenetic signatures reveal multilevel selection and fitness costs in SARS-CoV-2. https://doi.org/10.5281/zenodo.10522713 ^31^. This project contains the following underlying data: ExtendedData.pdf supplementary text, figures, and table cited in the paper ExtendedData_FileS1.txt - output of Poisson regressions across the ten employed cluster thresholds for the time period before Omicron BA.1.* emergence (early June 2020 to mid-November 2021) that tested whether TFPs were enriched in particular genomic regions or major PANGO lineages. ExtendedData_FileS2.txt - output of Poisson regressions across the ten employed cluster thresholds for the time period including Omicron BA.1.* emergence (early June 2020 to the end of April 2022) that tested whether TFPs were enriched in particular genomic regions or major PANGO lineages. ExtendedData_FileS3.zip - this ZIP file contains other four files: sc2_md_curated_WITH_Xs_Ns.rds - underlying preprocessed metadata file to use as input for mlscluster to reproduce the analysis. sc2_tre_curated.rds - underlying preprocessed time-scaled phylogenetic tree file to use in combination with the metadata file as input for mlscluster to reproduce the analysis. res_p2.rds - output of a time-consuming run of the mlsclust function ( https://github.com/mrc-ide/mlscluster/blob/main/R/mlsclust.R) for the period excluding Omicron (June 2020 to mid-November 2021). res_p3.rds - output of a time-consuming run of the mlsclust function for the period including Omicron (June 2020 to April 2022). Data are available under the terms of the Creative Commons Attribution 4.0 International license (CC-BY 4.0). Analysis code available from: https://github.com/vinibfranc/mlscluster-experiments Archived source code at time of publication: https://doi.org/10.5281/zenodo.10520060 ^25^ License: MIT

## Software availability

We implemented the inference methods described in this paper into a new R package entitled mlscluster Source code available from: https://github.com/ mrc-ide/mlscluster

Archived source code at time of publication: https://doi.org/10.5281/zenodo.10523291 ^23^

License: MIT
