# Phylogenetic signatures reveal multilevel selection and fitness costs in SARS-CoV-2

**DOI:** 10.12688/wellcomeopenres.20704.2

**Published:** 2024-07-24

**Authors:** Vinicius Bonetti Franceschi, Erik Volz

**Affiliations:** 1 Department of Infectious Disease Epidemiology, School of Public Health, Imperial College London, London, England, W2 1PG, UK

**Keywords:** Molecular evolution, phylogenetic analysis, transmission fitness, natural selection, mutation, genetic clustering, within-host evolution, SARS-CoV-2

## Abstract

**Background:**

Large-scale sequencing of SARS-CoV-2 has enabled the study of viral evolution during the COVID-19 pandemic. Some viral mutations may be advantageous to viral replication within hosts but detrimental to transmission, thus carrying a transient fitness advantage. By affecting the number of descendants, persistence times and growth rates of associated clades, these mutations generate localised imbalance in phylogenies. Quantifying these features in closely-related clades with and without recurring mutations can elucidate the tradeoffs between within-host replication and between-host transmission.

**Methods:**

We implemented a novel phylogenetic clustering algorithm ( mlscluster, https://github.com/mrc-ide/mlscluster) to systematically explore time-scaled phylogenies for mutations under transient/multilevel selection. We applied this method to a SARS-CoV-2 time-calibrated phylogeny with >1.2 million sequences from England, and characterised these recurrent mutations that may influence transmission fitness across PANGO-lineages and genomic regions using Poisson regressions and summary statistics.

**Results:**

We found no major differences across two epidemic stages (before and after Omicron), PANGO-lineages, and genomic regions. However, spike, nucleocapsid, and ORF3a were proportionally more enriched for transmission fitness polymorphisms (TFP)-homoplasies than other proteins. We provide a catalog of SARS-CoV-2 sites under multilevel selection, which can guide experimental investigations within and beyond the spike protein.

**Conclusions:**

This study provides empirical evidence for the existence of important tradeoffs between within-host replication and between-host transmission shaping the fitness landscape of SARS-CoV-2. This method may be used as a fast and scalable means to shortlist large sequence databases for sites under putative multilevel selection which may warrant subsequent confirmatory analyses and experimental confirmation.

## Plain Language Summary

Viral mutations can potentially carry a transient advantage, being simultaneously favourable for replication within hosts (e.g. by evading host immune responses) and deleterious to transmission (e.g. by having reduced cell binding). To identify such mutations, called transmission fitness polymorphisms (TFPs), we developed a clustering algorithm entitled mlscluster that computes clade-level statistics based on the number of descendants, persistence times, and growth rates of clades carrying a specific mutation in comparison with their immediate sisters without the mutation, which usually are different than expected in the presence of such TFPs. We then applied it to a representative SARS-CoV-2 time-scaled tree with >1 million whole-genome sequences from England.

Our statistical analysis suggested approximately constant levels of transient selection across waves driven by very distinct variants. It also showed that genomic regions of known functional significance such as spike, nucleocapsid, and ORF3a were enriched for TFPs. This is the one of the first studies to characterise SARS-CoV-2 recurrent mutations potentially under multilevel selection, providing empirical evidence for the existence of important tradeoffs in selection between intrahost replication and inter-host transmission. Therefore, it provides target mutations for realistic coalescent-based modelling and laboratory-based investigations of their impacts and mechanisms of interaction with human cells.

## Introduction

Although the majority of polymorphic sites within a viral genome are selectively neutral or under weak selection ^1^, frequent variant replacements driven by adaptive evolution are observed in SARS-CoV-2, Influenza A, and other endemic viruses ^2^. Despite less understood, the phenomena where some mutations confer a large transient increase in fitness, being advantageous to viral replication within hosts but detrimental to transmission, could present large epidemic-level effects. Well-documented examples of multilevel selection are provided in the context of HIV-1, which evolves considerably faster within individuals than at the epidemic level ^3, 4^, and virus which is more highly diverged from the population consensus is less likely to be transmitted ^5^. A variety of mechanisms may underlie multilevel selection effects, including preferential transmission of ancestral variants ^4^, switch in coreceptor usage ^6^ or heterogeneous immune selection and frequent reversions of host-specific adaptations ^7^. Human Influenza A is an endemic virus that evolves more similarly to SARS-CoV-2. Background selection outside, and not only positive selection within, antigenic epitopes was shown to control the mode and rate of influenza evolution, as immunological adaptation and the preservation of other viral functions can conflict with one another ^8^. 

During the COVID-19 pandemic, analyses of the extensive genomic data collected provided valuable insights about the competing forces influencing SARS-CoV-2 evolutionary dynamics ^9– 14^. However, it also highlighted the need for more advanced methods able to simultaneously handle large-scale datasets and perform fine-scale inferences (e. g. investigating fitness cost of individual mutations) ^9, 15– 17^. By developing such scalable analytic pipelines, we are able to identify recurrent mutations in different branches of a phylogenetic tree from a densely sampled epidemic, which potentially arise convergently as a consequence of virus response to adaptive selective pressures within hosts.

 The inference of fitness from the shape of phylogenies was introduced through the local branching index (LBI) ^18^ and treeImbalance methods ^19^. Remarkably, even when clades are distantly related, they can present very similar distributions of coalescent times and branch lengths ^20^, as well as the proportion of descendants, persistence time (or longevity), and growth rates when compared with closely-related clades. Most importantly, mutations influencing virus transmission fitness are expected to affect the distribution of offspring ^9^, consequently generating localised and quantifiable imbalance in time-scaled phylogenies. Therefore, the comparison of these parameters can indicate if similar evolutionary, demographic, or epidemiological processes are shaping viral evolution across different clades of a phylogenetic tree.

In molecular epidemiological studies, a set of particularly scalable approaches have been developed based on the detection of phylogenetic clusters comprising two or more closely related samples ^21, 22^. The frequency of phylogenetic clustering in a sample is sometimes considered a proxy for high transmission rate, especially in HIV datasets ^21, 23, 24^, and can potentially indicate spread efficiency of a particular genotype (e.g. HIV drug resistance-associated mutations [DRAMs]). Intuitively and by extension, transmissibility and within-host evolution between variants can be considered a proxy for overall fitness ^25^. Recently, a genetic clustering analysis of HIV-1 identified variants containing specific DRAMs in antiretroviral therapy (ART)-naive transmission networks that reduce transmission fitness and suggested a negative correlation between lower frequencies of rare polymorphisms and fitness advantage ^24^.

Currently, similar clustering approaches have not yielded major insights into negatively-selected variants in SARS-CoV-2 despite the collection of unprecedented numbers of whole-genome sequences ^9^. Furthermore, there is considerable scope to improve on distance-based genetic clustering methods because such approaches were demonstrated to be systematically biased to detect variation in sampling rates instead of transmission rates ^22^. Consequently, these methods will potentially present poor specificity for variants that negatively influence fitness. During the past few years, positive and negative selection in SARS-CoV-2 have mainly been investigated using methods that rely on synonymous rate variation across sites/branches ^26, 27^, and results from these approaches on SARS-CoV-2 comprehensive datasets are available for comparison ^28^. However, methodology to identify mutations that potentially have a transient fitness advantage is still lacking.

We developed a tree-based clustering algorithm, available as open-source R package mlscluster () ^29^, to identify potential transmission fitness polymorphisms (TFPs), which can be defined as mutations carrying a transient/multilevel fitness advantage, i.e. being simultaneously beneficial for replication within hosts and deleterious to transmission. This is achieved by computing and comparing simple summary statistics from the offspring of recurring clade-defining mutations in a time-scaled phylogeny, therefore specifically investigating homoplasies and aggregating information across many occurrences of the same mutation. This approach complements standard procedures based on synonymous rate variation across sites/branches by highlighting variants which likely have different and/or competing selective pressures within and between hosts. We demonstrate its applicability through the analysis of a representative SARS-CoV-2 data set comprising >1.2 million genome sequences from England. The analysis indicated slightly higher TFP-homoplasy enrichment on B.1.1.7 and AY.4.* lineages, particularly in genomic regions of known functional significance such as spike (S), nucleocapsid (N), and ORF3a, as well as regions of poorly understood significance including ORF7 and ORF8. By providing a comprehensive catalog of the main sites potentially resulting from multilevel selective pressures throughout the SARS-CoV-2 genome, we also expand the understanding of the SARS-CoV-2 fitness landscape outside the well-studied spike protein. Therefore, these results are meant to guide the design of experimental studies and population genetic models to characterise the functional impact of specific mutations, especially the subset that is advantageous to within-host replication but detrimental to transmission.

## Methods

### Terminology and tree-based clustering statistics for detecting localised imbalance

**Figure 1.**
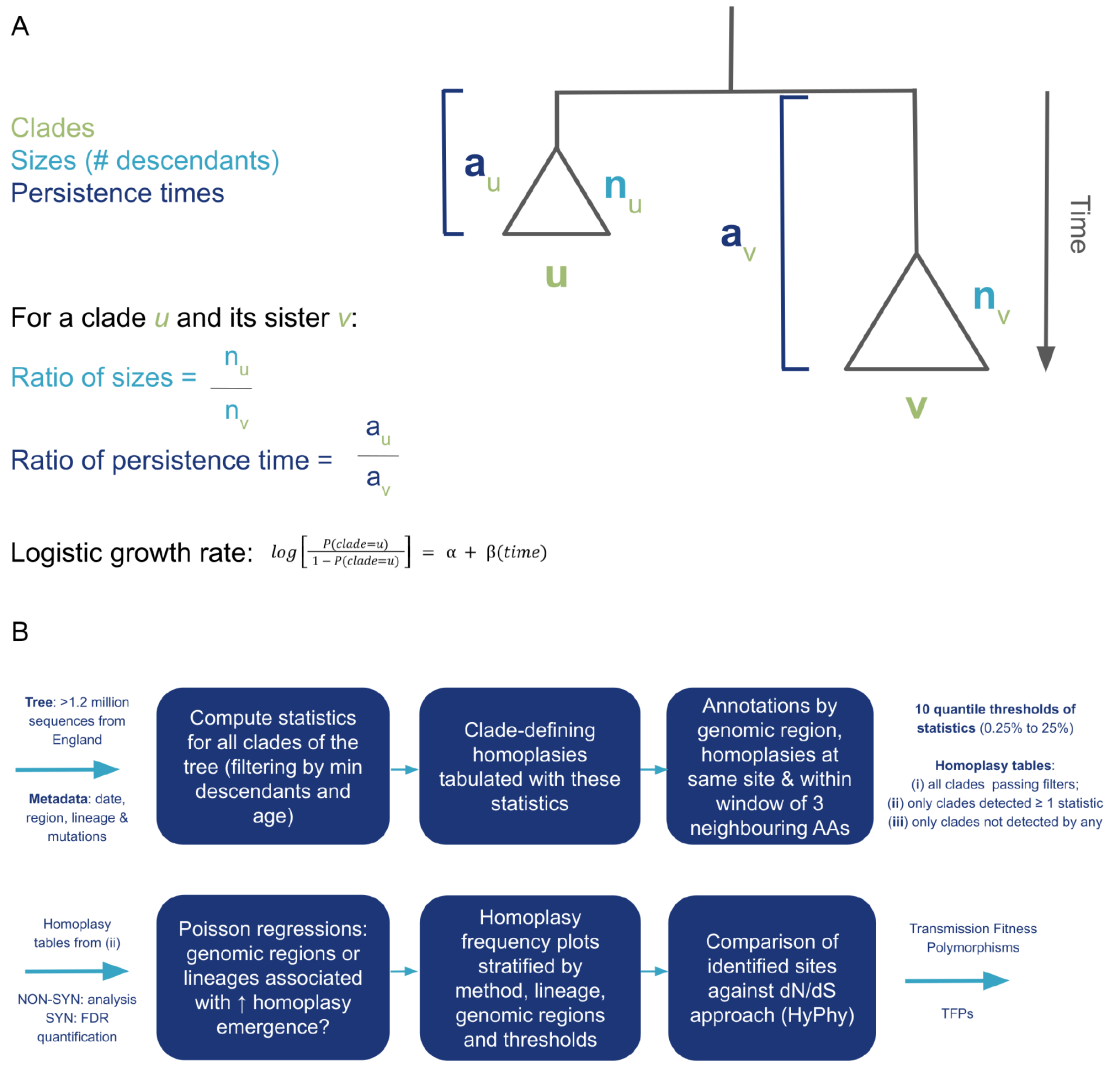
Schematic view of the tree-based clustering algorithm implementation and analytic pipeline. ( **A**) Main notations, parameters, and respective statistic formulas that are computed by mlscluster ( https://github.com/mrc-ide/mlscluster) for sister clades of the time-scaled phylogeny. ( **B**) Analysis workflow with main steps from input data to TFP inference.

We propose a phylogenetic tree-based clustering algorithm to systematically explore all clades in a time-scaled phylogenetic tree reconstructed from viral genomes. Assume two clades *u* (target clade) and *v* (comparator/sister) ( Figure 1A) organised in a time-scaled tree *t* and sharing full ancestry ( *i.e.*, the same defining mutations) up to the point which they diverge and present their own exclusive defining mutations. The size of each clade ( *n* in Figure 1) is defined as the number of descendant sequences arising from it until the leaves of the tree are reached. The persistence time (given by *a* in Figure 1), is defined as the difference between the maximum sample time of samples descended from that clade and the estimated time of its most recent common ancestor [tMRCA]. After computing these simple parameters, the target clades *u* are then contrasted against their comparators *v*, which can be their sister clade (the onesharing an immediate ancestor assuming bifurcating phylogenetic relationship) or all other clades sharing the same immediate ancestor (in case of polytomies/multifurcations).

If considering a clade *u* with sister clade *v*, we compute three statistics based on these local phylogenetic patterns: (i) the ratio *S _uv_* of ‘clade sizes’ (i.e., the number of samples descended from each clade) between *u* and *v* denoted *n _u_* and *n _v_* ( Equation 1), (ii) the ratio *T _uv_* of persistence times (i.e., longevities) denoted by *a _u_* and *a _v_* ( Equation 2), and (iii) the logistic growth rate. The latter is defined as the coefficient of a logistic regression having a response variable defining sampling a descendent of *u* versus *v* and the sample time as a predictor ( Equation 3). The coefficient of such a logistic regression quantifies the relative growth of the clade and is related to the selection coefficient ^30^.


$$\;S_{uv}\;=\;\;\frac{n_{u}}{n_{v}}\;(1)$$

$$\;T_{uv}\;=\;\;\frac{a_{u}}{a_{v}}\;(2)$$

$$\log\;\left[\frac{P(\text{clade}\;=\;\text{u})}{1–P(\text{clade}\;=\;\text{u})}\right]\;\;=\;\;\alpha \;\;+\;\;\beta (\text{time})\;(3)$$

### Tree-based clustering algorithm implementation

The mlscluster method is implemented as an R package ( https://github.com/mrc-ide/mlscluster) ^29, 31^ that incorporates these multiple statistical methods for identifying especially convergently acquired mutations (homoplasies) that are potentially detrimental for transmission (i.e., homoplasies occurring in a subtree for which at least one of the three aforementioned statistics fall below a low quantile [e. g., 2%] of the empirical distribution). These statistics applied to each clade are designed to be simple and computationally fast, making it possible to scan phylogenies with more than a million tips in hours using multiple CPU cores.

The clustering algorithm ( Figure 1B) starts by receiving a rooted bi- or multifurcating time-scaled tree ( *e. g.*, estimated using treedater ^32^, treetime ^33^ or chronumental ^34^) and associated metadata in a tabular format including sequence name, sample date, lineage, major lineage, and annotated mutations. The package then uses standard tree manipulation strategies implemented in the ape R package ^35^, particularly postorder traversal to visit clades and tips based on the two-column edge matrix from the “phylo” class. Given this efficient way to visit clades of the tree and edge lengths, we can easily extract the parameters of interest ( *e. g.*, time of the most recent common ancestor of each clade, descendant identifiers and quantities, clade ages, etc). Therefore, target clades are selected if they meet all of the following conditions: (i) have at least a specified minimum number of descendants (ii) have up to a maximum number of descendants, (iii) last for a minimum period (cluster age, in years), (iv) are sampled after a minimum sampling date, and (v) are sampled up to a maximum sampling date. The default values of (i) 10, (ii) 20×10 ^-3^, and (iii) 1/12 (1 month) were chosen to avoid ratios being taken from small or unreliable clade sizes/persistence times, an unrealistically high number of viral generations, and very short timeframes. These values were selected based on previous experience with UK SARS-COV-2 analyses using a similar software developed by our research group ^15, 36^. A systematic evaluation of how these parameters would influence the results was not performed, but is planned for future applications of this method. The minimum date was set to be the one of the Wuhan reference sequence and the maximum dates were modified according to the investigated pre-Omicron (up to mid-November 2021) and including Omicron (up to end of April 2022) periods. 

Subsequently, target and comparator (sister) clade(s) are gathered together and ratio of sizes, ratio of persistence time and logistic growth rates are calculated as previously stated. Since every sequence should include a metadata column (e.g. precomputed by COG-UK consortium, see ) listing mutations from its genome, the clustering algorithm tool incorporates a function to identify defining polymorphisms in target clades. The mutation must be present in >75% of the sequences in that clade (while absent or in a smaller fraction than this percentage in its comparator) to be considered as defining, although this cutoff value can be changed. After computing clade-defining mutations, these are all tabulated and those which happen more than once in different clades are retrieved as homoplasies. To enhance inspection of results, homoplasies are annotated into (i) regions of interest ( *e.g.* SARS-CoV-2 spike and nucleocapsid proteins), (ii) different mutations at the same site, and (iii) mutations within a 3 amino acid sliding window. There is also an additional sanity check for known sequencing artifacts ^37^ and for positively selected sites found by other analyses ^28^. Then, based on cut points dividing the range of the empirical distribution of each statistic into equally-spaced continuous intervals (quantiles), cluster thresholds can be specified. These thresholds retain only clades, along withtheir respective defining homoplasies, that are potentially detrimental for transmission (default threshold of <1%) or carrying a positive fitness advantage (e.g., >99%), therefore aggregating summary statistics from the phylogeny across many occurrences of the same mutation when compared with sister clades without the mutation. We intended to make the method flexible by creating a parameter that specifies in how many percentiles the statistic should be splitted ( *default* = 1/100) and another to keep values below or above the cutoff point.

Different comma-separated detailed outputs are generated for each of the three statistics showing clades (and defining homoplasies) contained in the chosen cluster threshold, as well as the intersection (the ones identified by the threshold of the three statistics) and union (clades associated with at least one statistic threshold). Additionally, for each of the three homoplasy-annotated categories, three files are generated with their frequencies, and clades of occurrence considering (a) all target clades that passed minimal filtering conditions, (b) only clades detected by one or more statistic threshold, (c) the ones not detected by any cutoff. Finally, these outputs are joined into one *data.frame* to facilitate further statistical analyses.

### Identifying potential TFPs using the mlscluster algorithm and a representative SARS-CoV-2 genomic dataset

***Tree and metadata.*** A global SARS-CoV-2 maximum likelihood (ML) phylogenetic tree was estimated using FastTreeMP v2.1.11 ^38^ and UShER ^39^ as part of the phylopipe workflow ^40^. In addition, associated metadata including adm2 regions following the Database of Global Administrative Areas (GADM) subdivisions, PANGO-lineages, and annotated synonymous and non-synonymous mutations were obtained from the COVID-19 Genomics UK Consortium (COG-UK). From the ML tree, we estimated a time-scaled phylogeny using chronumental v0.0.53 ^34^. Only sequences from England were retained alongside the Wuhan/WH04/2020 (EPI_ISL_406801) reference sequence. In total, we included 1,275,669 sequences from all the 118 adm2 regions in England from June 01, 2020 to April 30, 2022, since they are associated with Pillar 2 representative community sampling efforts in the UK.

**Figure 2.**
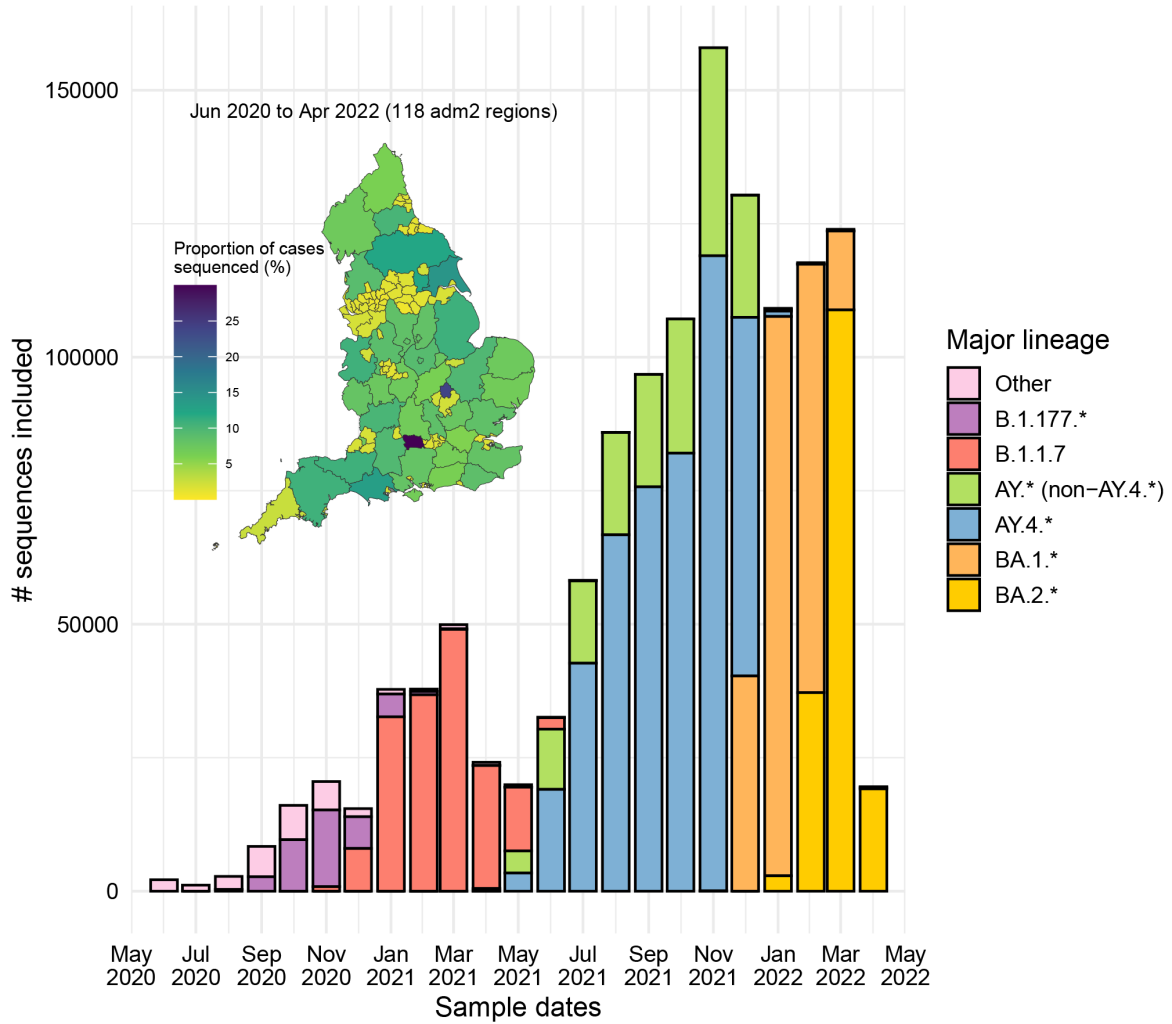
Spatiotemporal distribution of the SARS-CoV-2 sequences from England included in this study during the investigated period (June 2020 to April 2022). Main plot: Monthly-stratified frequency of the sequences stacked by major PANGO-lineage. Inset plot: Proportion of included sequenced cases across adm2 regions in England during the investigated period for 77% of the samples with unambiguous adm2-level assignments.

The proportion of cases sequenced for each region in England was computed by using Upper Tier Local Authorities (UTLA) and Lower Tier Local Authority (LTLA)-level case counts obtained from the UK government website ( https://coronavirus.data.gov.uk/, accessed on 27 April 2023) matched against GADM adm2 geographical regions contained in COG-UK metadata. Since adm2, LTLA and UTLA regions are not entirely compatible, we have not considered on sequence counts samples with ambiguous matches (33%) for the map representation ( Figure 2).

### Statistical analysis for identifying genomic regions enriched for TFPs

We tested our approach using two COVID-19 pandemic time-periods: (i) from June 01, 2020 (including Wuhan/WH04/2020 reference sequence as root of the phylogeny) to November 15, 2021 (before Omicron BA.1.* variant emergence) (ii) from June 01, 2020 to April 30, 2022 (considering Omicron BA.1.* and up to Pillar 2 termination). For each period, 10 different thresholds (0.25%, 0.5%, 0.75%, 1%, 2%, 3%, 4%, 5%, 10%, and 25%) of the clustering statistics are considered.

We also performed rigorous quality control to ensure our estimates were not biased by sequencing and base-calling artifacts. Firstly, we removed outlier sites highly enriched for homoplasies (above the 99% quantile of homoplasy frequencies for all cluster thresholds), which we manually confirmed to be sequencing artifacts due to the high number of undetermined bases at respective sites in the alignment generating the phylogenetic tree. However, even after performing this approach, BA.1-defining mutations in the Receptor-binding Domain (RBD) were particularly identified as TFP-homoplasies for threshold=2% (Extended Data Figure S1A) ^41^, which was an unexpected result. To further inspect this inconsistency, we selected eight BA.1-spike defining mutations that were in our top100 of most frequent homoplasies (S:S371L, S373P, S375F, G496S, Q498R, N501Y, Y505H, N764K) and excluded all sequences (n=71,414, 5.6% of all sequences) that had undetermined bases (e.g. "NNN") in their respective codons from the nucleotide alignment. As a result, these sites were not detectable anymore (Extended Data Figure S1B) ^41^ and we could confirm they were the result of sequencing artifacts, which generally occur due to systematic differences in sequencing protocols and primer selection over time ^42^ and in different laboratories.

Consequently, we decided to perform a more aggressive quality control (henceforth called alignment-aware artifact removal) that has the advantage of not relying on excluding sequences without perfect coverage. First, we ran the mlscluster algorithm ( https://github.com/mrc-ide/mlscluster) without any artifact removal. We then extracted every homoplasic site detected and used seqtk v1.3-r106 ^43^ to create alignment files for each codon matching these sites in case of non-synonymous mutations and for each nucleotide in case of synonymous mutations. Afterwards, for each sequence, we added "X" (undetermined amino acid) for every site which had one or more non-ACTG character in respective codon positions, and "N" (undetermined nucleotide) for every non-ACTG synonymous site. Subsequently, we appended the "X" and "N" site annotations for all sequences within the existing metadata mutations column. We then incorporated a function named .fix_sites ( https://github.com/mrc-ide/mlscluster/blob/main/R/mlsclust.R#L314) to deal with those highly uncertain sites within the mlscluster package. In summary, since the first step to extract a defining mutation for each target clade is to compute the frequency of each mutation within the target and the comparator clades, we used the proportion of the most frequent mutation at a given site across all sequences within the clade and added up the frequency of the "X" or "N" at that site, because it is most likely that the artifacts follow the majority within that specific clade given the complete shared ancestry. For example, if the target clade has the site S:G446 changing to S with frequency = 0.7 (i.e., 70% of the sequences in the target have this mutation) and to X with frequency = 0.3, we consider the S frequency = 1, and this mutation now has enough frequency (>75%) to be considered defining, which only would occur if the comparator has S:G446S at frequency <0.75%. In cases where "X" and "N" are the most frequently mutated characters within a clade, the second-ranked amino acid or nucleotide at that site is added to these undetermined characters. For example, the target clade has the site S:N501 changing to X with frequency = 0.6 and to Y with frequency = 0.4, then the X frequency = 1. In such cases where either the target or comparator has a higher frequency of "X" or "N", the sites are not considered defining. A mutation is only considered defining when (i) it has one of the 20 valid amino acids (or stop codon) or the four valid nucleotides, (ii) it has a >75% frequency on the target clade and simultaneously <75% on the its comparator. This approach removed not only the eight previously investigated BA-1-spike defining mutations but also other six S sites for threshold = 2%, and affected mostly the BA.1 lineage without major changes for other lineages. Therefore, we consider that results arising from this alignment-aware artifact removal method are more reliable than previously and report those throughout the paper.

We performed Poisson regressions using the glm function from the stats package ^44^ and having the frequency of homoplasies as response variable ( Equation 4) to identify if any genomic regions and/or major PANGO-lineages were associated with increased TFP-homoplasy emergence for non-synonymous polymorphisms across different time periods and thresholds. A p-value ≤ 0.05 was considered statistically significant.


$$\begin{array}{l} \text{Freq}\_{\mathrm{homopl}}_{i\;}\sim \;\;\mathrm{Poisson}\;\left(\lambda_{i}\right)\\ \;\text{log}\;\left(\lambda_{i}\right)\;=\;\alpha_{j[i]}\\ \;+\;\beta_{1j[i]}(\text{major}\_\text{lineage})\\ \;+\;\beta_{2j[i]}(\text{genomic}\_\text{region})\\ \;+\beta_{3}(\text{indep}\_\text{positive}\_\text{selection})\;(4) \end{array}$$

We assigned as major PANGO-lineages the following variants: B.1.177, Alpha (B.1.1.7), Delta AY.4 and sublineages (AY.4.*), other Delta (AY.* [non-AY.4.*]), Omicron BA.1.*, Omicron BA.2.*, and Others (all other lineages excluding recombinants). These were main drivers of epidemic waves in the UK and around the globe. Genomic regions included all 15 non-structural proteins (NSPs) from ORF1ab (NSP1-10, 12-16), S, ORF3a, Envelope (E), Membrane (M), ORF6, ORF7a, ORF7b, ORF8, N, and ORF10. Moreover, regions of characterised functional significance including the N-terminal Domain (NTD), the RBD and the Furin-cleavage site (FCS) of S, as well as the Linker Domain of N ^45^ were considered as additional genomic regions. The other covariate was whether the sites were independently found under selection based on a HyPhy-based synonymous rate variation across sites/branches analysis ^28^.

To further investigate the genomic regions enriched for TFP-homoplasies resulting from the Poisson regressions and to compare the sites identified as potentially under selection against results from the literature ^28^, we generated different exploratory visualisations using ggplot2 ^46^. These were stratified by the method of detection ( mlscluster, HyPhy, or both), major PANGO-lineages, genomic regions (including frequency normalisation by size), and cluster thresholds.

Although not strictly comparable, we also examined how our estimates of multilevel selection were consistent with deep-mutational scanning (DMS) experiments ^47– 51^ and comprehensive computational estimates of the magnitude of one level of selection based on fixed fitness effects ^52^, Firstly, we extracted DMS measurements against spike ^47^, RBD ^48^, and NSP5 (MPro) ^49– 51^ as compiled previously ( https://github.com/jbloomlab/SARS2-mut-fitness/tree/main/results/dms). Subsequently, we selected fitness effects estimates ^52^ that would be suitable for comparison against our dataset, leading to the choice of the England subset from early 2022 ( https://github.com/jbloomlab/SARS2-mut-fitness/blob/main/results_public_2022-01-31/aa_fitness/aamut_fitness_by_subset.csv). We then matched mutations detected as TFPs and non-TFPs (homoplasies that are not identified by any of the mlscluster statistics) against mutations having positive and negative effects given by the respective DMS (experimental) and computational estimates. We also compared the association between TFP status and fitness magnitude using a Fisher's Exact test. Finally, we explored how the distribution of computational selection coefficients was different for TFPs and non-TFPs using sina plots and a Wilcoxon signed-rank test.

### Codon-aware false discovery rate (FDR)

We used synonymous homoplasies for characterising the FDR of our approach under the assumption that synonymous sites would not provide a fitness advantage (i.e., are nearly neutral, as demonstrated ^52^). Since these sites represent one-third of the genome and mutations tend to occur in the third codon position to preserve the encoded amino acid, this weighting needs to be taken into account when computing FDR. Firstly, we defined the percentage of erroneous TFP calls for each threshold *t* as:


$$\;{\mathrm{FDR}}_{t}\;=\;\;\frac{Y_{t}}{i}\;\;\times \;\;100\;(5)$$

where *Y* is the number of TFP calls specifically among the *i* polymorphic third codon position sites with > 100 mutated sequences at the given site (considering the analysed > 1.2 million genomes). This threshold was selected after retrieving the number of polymorphic synonymous sites at each codon position for thresholds ranging from 10 to 1000 mutated sequences, balancing the number of legitimate and possibly artifactual polymorphic sites and representing approximately 0.1% of the analysed sequences. The FDR calculation is also performed for each SARS-CoV-2 protein. Multiplication by 100 transforms the proportion of erroneous TFP calls into a percentage for easier interpretation.

Similarly, a separate error rate ( *ε* or codon-aware FDR) is also computed relative to the sites at first and second codon positions as follows:


$$\;\varepsilon_{t}\;=\;\;\frac{{\mathrm{FDR}}_{t}\times \;\;Z_{t}}{j}\;\;(6)$$

where *Z* is the number of TFP calls at codon positions one and two, and *j* is the total number of polymorphic sites at first and second positions with > 100 mutated sequences at the given site.

Both calculations were performed for the two analysed time periods, with slightly smaller error rates for the timeframe before Omicron emergence.

## Results

We utilised our new approach ( Figure 1) ^41^ to analyse 1,275,669 SARS-CoV-2 whole genome sequences from England sampled between June 2020 and April 2022. This time period encompasses: (i) a period from June to December 2020 dominated by A.*, B.* and B.1.177 lineages, (ii) a timeframe between January and May 2021 when Alpha (B.1.1.7) predominated, (iii) a wave from June to December 2021 characterised by rapid spread of Delta (AY.4.* and other AY.*), and (iv) the Omicron (BA.1 and BA.2) epidemic cycle from December 2021 to April 2022 ( Figure 2). For clarity and due to the main patterns observed, the analytic period presented includes: (i) June 2020 to mid-November 2021 (pre-Omicron interval) and (ii) mid-November 2021 to April 2022 (including Omicron). Only data collected through community sampling (Pillar 2) were included to reduce bias towards more severe infections and avoid the inclusion of data that was collected for special purposes. For these reasons, we were unable to investigate e.g. second-generation BA.2, BA.5, XBB, and other relevant variants that emerged after April 2022. The geographical representation of the data is similar across different regions of England, with a mean proportion of community cases having a sequence of 6.7% and median of 7.1% ( Figure 2, inset plot).

### Lineages and genomic regions enriched with SARS-CoV-2 TFP-homoplasies

**Figure 3.**
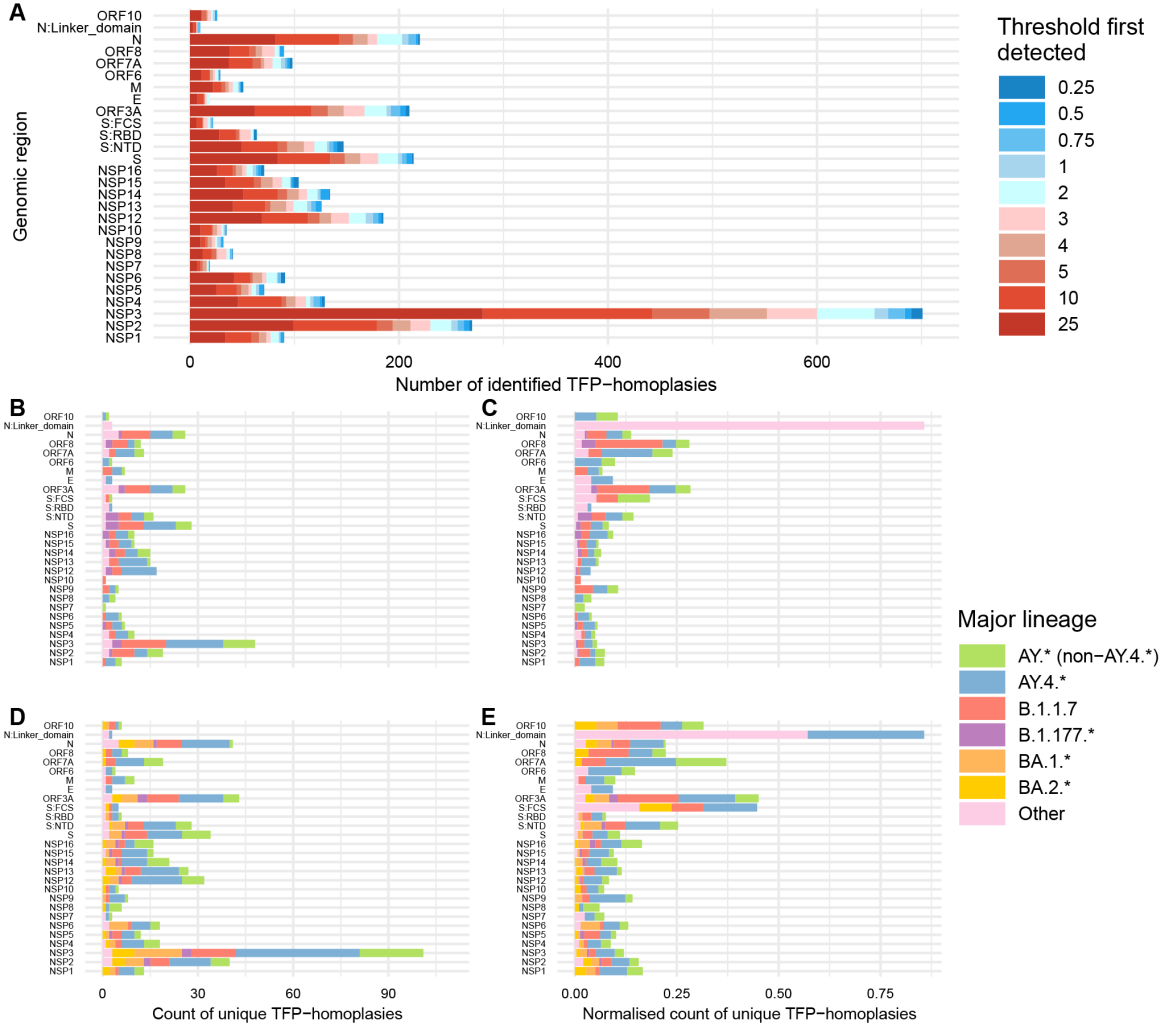
Frequency of SARS-CoV-2 TFP-homoplasies per genomic region considering all cluster thresholds and the more reliable threshold of 2%. ( **A**) Count of TFP-homoplasic sites for all SARS-CoV-2 proteins across the 10 different cluster thresholds ranging from the more (0.25%) to the less stringent (25%). ( **B**– **E**) Count of TFP-homoplasies per genomic region for two different time periods and considering threshold = 2%. ( **B**) Non-normalised counts per lineage for timeframe pre-Omicron (June 2020 to mid-November 2021). ( **C**) Normalised counts per lineage (divided by genomic size) for the same period as ( **B**). ( **D**) Non-normalised counts for the timeframe including Omicron (June 2020 to end of April 2022). ( **E**) Normalised counts for the same period as ( **D**).

The presence of recurring synonymous polymorphisms classified as TFP-homoplasies allowed us to investigate the FDR for each genomic region as a function of the applied cluster thresholds. The frequency of synonymous mutations along the genome (Extended Data Figure S2) ^41^ and the FDR across genomic regions (Extended Data Figure S3) ^41^ support that sites only detected at thresholds ≥ 5% must be investigated with caution since they generally have associated FDRs >20% and *ε* ≈ 5%. In contrast, cutoffs ≤ 2% retain acceptable FDRs ≈ 10% and *ε* ≈ 2%. Additionally, these more erroneous thresholds (≥ 5%) represent > 50% of the identified TFP sites ( Figure 3A).

Therefore, among the ten cluster thresholds ranging from the more strict (0.25%) to the more lenient (25%) values (Extended Data Text S1, Extended Data Figure S4) ^41^, we report results with the 2% threshold and after performing rigorous quality control using an alignment-aware artifact removal method to represent sites under putative multilevel selection (see Methods: Statistical analysis for identifying genomic regions enriched for TFPs). With this threshold, the false discovery rate (FDR) and codon-aware FDR ( *ε*) (see Methods: Codon-aware false discovery rate (FDR)) are respectively around 10% and 2.5% (Extended Data Figure S3) ^41^. Results for sites identified with other thresholds are presented in the Extended Data ^41^.

For the period predating Omicron BA.1.* emergence, we found that B.1.1.7 was consistently the lineage with the highest coefficient for enrichment of TFP-homoplasies, reaching statistical significance for ≥ 2% thresholds. However, TFP enrichment was similar across lineages and not found for specific genomic regions (Extended Data File S1) ^41^. Although not significantly different, TFP-homoplasies were slightly more abundant in the small linker domain ^45^ of the N protein, ORF3a, and ORF8 for this time period ( Figure 3B and C).

All considered major lineages were associated with increased TFP-homoplasy emergence for ≥ 1% thresholds during the timeframe which includes Omicron BA.1.* as the dominant variant. Although not consistent for different thresholds, the rank of lineage coefficients was Other lineages > B.1.1.7 > AY.4.* > BA.1.* > AY.* (non-AY.4.*) for threshold = 2%. Once again, there were no statistically significant results at the 2% threshold regarding genomic regions (Extended Data File S2) ^41^. However, N:linker domain, ORF3a, S:FCS, ORF7a, and ORF10 presented a higher number of TPFs per site ( Figure 3D and E), which is relatively consistent with the preceding period.

Normalising homoplasy counts by the size of each genomic region (giving less weight to larger genomic regions) has a large influence in interpreting the relative rates of TFP acquisition. This is especially demonstrated by NSP3, which accrues more TFPs due to its size of 5835 nucleotides ( Figure 3B and D), but when normalised has a similar distribution of TFPs compared to other genomic regions ( Figure 3C and E).

### TFPs along the SARS-CoV-2 genome and low concordance with positively selected sites

**Figure 4.**
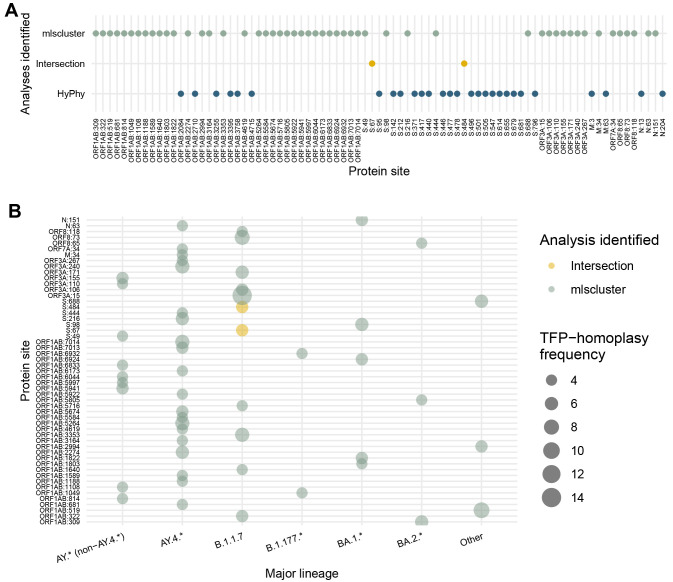
TFP-homoplasy identification compared to sites identified as under positive selection. Sites are compared across different major lineages. ( **A**) Comparison of top 30 identified sites under multilevel selection by our tree-based clustering approach for the whole-period (including Omicron) for cluster threshold = 2% against the HyPhy analysis ^28^, also presenting concordant results (intersection) between both methods. ( **B**) Bubble plot of TFP-homoplasy frequencies attributed to different major PANGO-lineages. The HyPhy analysis only contained the identified sites and not the underlying amino acid replacements. The actual mutations and further annotations are presented in Table 1.

When comparing the 30 most frequent mlscluster TFP-homoplasies against the 30 mutations under positive selection detected by the HyPhy-based approach ^28^ for the cluster threshold = 2% and period before Omicron emergence, we detected three concordant sites (S:67, S:95, and S:484), 27 positively selected sites only detected by HyPhy, and 63 sites only detected by mlscluster statistics (Extended Data Figure S5) ^41^. For the timeframe including Omicron, we identified two sites under selection concordantly between methods and with the previous period (S:67 and S:484), 28 discordant results, and 51 new potential TFPs across seven proteins/ORFs ( Figure 4A). Notably, the minimal overlap between sites identified as under multilevel ( mlscluster) and positive (HyPhy) selection suggests these methods are indeed capturing different selective pressures, as expected.

The top 30 most frequent TFP-homoplasies across lineages shows that the B.1.1.7 (n=22) and the AY.4.* (n=21) were similarly enriched for those highly-frequent TFP-homoplasies up to mid-November 2021 (Extended Data Figure S5) ^41^, while AY.4.* (n=20) was notably the major lineage harbouring more TFPs ( Figure 4B, Table 1) when considering Omicron BA.1.*. Although this would technically mean that these lineages are morely likely to give rise to TFPs, it is possible that this observation is driven by surveillance effects, since there was relatively deep sampling of B.1.1.7 and AY.4 that may have enabled the detection of more candidate TFPs. Morever, the analysis of lineage-specific top 30 TFP-homoplasies regardless of threshold shows that less than half of those are firstly detected on smaller cluster thresholds (up to 2%) (Extended Data Figures S6–S10) ^41^.

**Table 1.** Top 30 TFP-homoplasies within the spike protein for the period between June 2020 and April 2022 (including Omicron) and cluster threshold = 2%.

Homoplasy	Frequency	Major lineage	HyPhy	Genomic region	Amino acid length of protein
ORF3A:L15F	14	B.1.1.7	No	ORF3A	275
ORF1AB:G519S	9	Other	No	NSP2	638
ORF8:Y73C	8	B.1.1.7	No	ORF8	121
ORF1AB:H5264Y	7	AY.4.*	No	NSP12	932
ORF1AB:K3353R	7	B.1.1.7	No	NSP5	306
ORF1AB:R7014N	7	AY.4.*	No	NSP16	298
ORF3A:P240S	7	AY.4.*	No	ORF3A	275
ORF1AB:P309L	6	BA.2.*	No	NSP2	638
ORF1AB:T2274I	6	AY.4.*	No	NSP3	1945
ORF3A:S171L	6	B.1.1.7	No	ORF3A	275
**S:A688V**	6	Other	No	S:FCS	38
**S:L216F**	6	AY.4.*	No	S:NTD	292
**S:S98F**	6	BA.1.*	No	S:NTD	292
N:P151L	5	BA.1.*	No	N	413
ORF1AB:A2994V	5	Other	No	NSP4	500
ORF1AB:K322R	5	B.1.1.7	No	NSP2	638
ORF1AB:L6924F	5	BA.1.*	No	NSP16	298
ORF1AB:P7013L	5	AY.4.*	No	NSP16	298
ORF1AB:S5674L	5	AY.4.*	No	NSP13	601
ORF1AB:T1822I	5	BA.1.*	No	NSP3	1945
ORF1AB:T5941I	5	AY.* (non-AY.4.*)	No	NSP14	527
ORF3A:D155Y	5	AY.* (non-AY.4.*)	No	ORF3A	275
ORF3A:L106F	5	B.1.1.7	No	ORF3A	275
**S:A67V**	5	B.1.1.7	Yes	S:NTD	292
**S:E484K**	5	B.1.1.7	Yes	S:RBD	223
M:L34F	4	AY.4.*	No	M	222
N:D63G	4	AY.4.*	No	N	413
ORF1AB:A1049V	4	B.1.177.*	No	NSP3	1945
ORF1AB:A5922V	4	AY.4.*	No	NSP13	601
ORF1AB:A6044V	4	AY.* (non-AY.4.*)	No	NSP14	527
ORF1AB:D5584Y	4	AY.4.*	No	NSP13	601
ORF1AB:G6173V	4	AY.4.*	No	NSP14	527
ORF1AB:H1108Y	4	AY.* (non-AY.4.*)	No	NSP3	1945
ORF1AB:L681F	4	AY.4.*	No	NSP2	638
ORF1AB:M5997I	4	AY.* (non-AY.4.*)	No	NSP14	527
ORF1AB:P1640S	4	B.1.1.7	No	NSP3	1945
ORF1AB:P1803S	4	BA.1.*	No	NSP3	1945
ORF1AB:P4619L	4	AY.4.*	No	NSP12	932
ORF1AB:P6932S	4	B.1.177.*	No	NSP16	298
ORF1AB:R3164H	4	AY.4.*	No	NSP4	500
ORF1AB:R5716C	4	B.1.1.7	No	NSP13	601
ORF1AB:S1188L	4	AY.4.*	No	NSP3	1945
ORF1AB:T1589I	4	AY.4.*	No	NSP3	1945
ORF1AB:T5805M	4	BA.2.*	No	NSP13	601
ORF1AB:T6833I	4	AY.* (non-AY.4.*)	No	NSP16	298
ORF1AB:T814I	4	AY.* (non-AY.4.*)	No	NSP2	638
ORF3A:A110S	4	AY.* (non-AY.4.*)	No	ORF3A	275
ORF3A:P267L	4	AY.4.*	No	ORF3A	275
ORF7A:P34L	4	AY.4.*	No	ORF7A	121
ORF8:A65V	4	BA.2.*	No	ORF8	121
ORF8:L118V	4	B.1.1.7	No	ORF8	121
**S:H49Y**	4	AY.* (non-AY.4.*)	No	S:NTD	292
**S:K444R**	4	AY.4.*	No	S:RBD	223

When expanding to the top 100 TFP-homoplasies (Extended Data Table S1) ^41^, 21 sites are located within the larger NSP3 (0.011 TFPs per site), 14 are from ORF3a (0.051 per site), 12 from spike excluding NTD, RBD, and FCS (0.017), nine from NTD (0.031), four from FCS (0.105), two from RBD (0.009), and seven from the N protein (0.017). Therefore, S:FCS and ORF3a presented the highest normalised frequency of TFP-homoplasies per site. Respectively, AY.4.* and AY.* account for 50 (36.5%) and 28 (20.4%) of these top 100 sites (that are actually 137 because multiple TFPs are tied with three observations), followed by B.1.1.7 (n=24, 17.5%). In summary, Delta lineages account for >50% of the identified top-ranked TFP homoplasies.

A manual inspection of TFP-homoplasies with frequency ≥ 5 (Extended Data Table S1) ^41^ confirmed that they emerged independently in multiple lineages during the pandemic and are predominantly found in extremely low (<1%) frequencies. This independent analysis shows that the method works as expected, capturing potential signals of multilevel selection. This is indicated by the recurrence of such mutations and their appearance in clades where the size, longevity or growth rate of the target clade is much smaller when compared to its sister(s). Additionally, it suggests the existence of potential functionally important mutations outside the spike protein that warrant further investigation. (Extended Data Table S1) ^41^.

**Figure 5.**
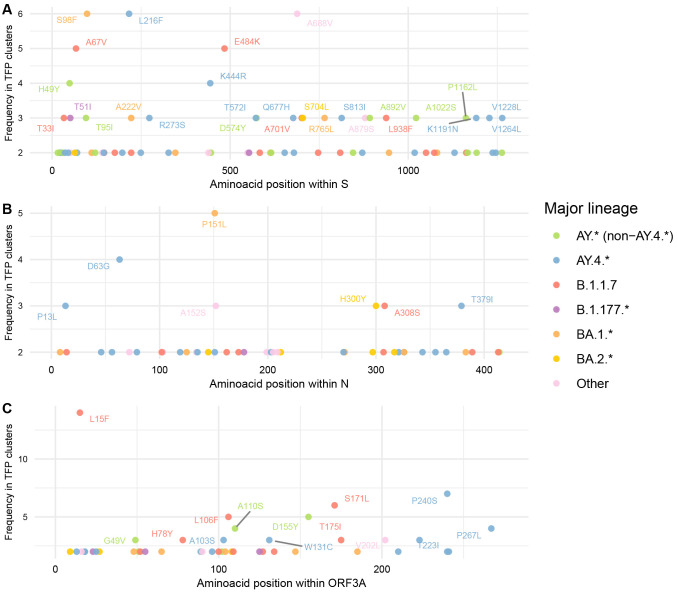
Frequency of identified TFP-homoplasies alongside genomic regions with major functional significance and normalised counts for cluster threshold = 2% and period including Omicron. ( **A**) Spike. ( **B**) Nucleocapsid. ( **C**) ORF3a. TFPs are coloured by major PANGO-lineage and annotated if frequency > 2.

By focusing on individual proteins that harbour a higher normalised count of TFPs and both established (S, N, ORF3a) and yet to be characterised functional significance (ORF7a and ORF8), we highlight relevant sites potentially under multilevel selection for further experimental investigations. These sites include S:A67V, S:S98F, S:L216F, S:E484K, S:A688V, N:P151L, ORF3a:L15F, ORF8:Y73C, etc. Additionally, these transient selective processes are more likely to be acting uniformly across each protein and not in specific hotspots ( Figure 5, Extended Data Figure S11, Extended Data Table S1) ^41^.

### Comparison against DMS experiments and other approaches to detect sites under selection

When exploring the DMS fitness effects for the spike protein ^47^, we identified a total of 49 TFP sites matching DMS negative effects and 15 sites corresponding to positive estimates (Extended Data Figure S12A). When tabulating these values with the non-TFPs (332 and 169, respectively), the resulting odds ratio of a TFP having negative fitness effect is 1.66 (95% CI: 0.88 to 3.29). The positive effect of such 15 TFPs is very close to zero (maximum: 0.35). Similarly, of the five TFPs within the RBD ^48^, four are associated with DMS small negative effects and one with a slightly positive effect (Figure S12B). On the other hand, our comparison against NSP5 (MPro) DMS experiments ^49, 50^, which greatly presented overall positive fitness effects (87.63% and 90.62% of all investigated mutations across these two studies), demonstrated that all 12 TFPs had small positive effects between zero and one. In another DMS study ^51^ that presented a much smaller percentage of positive effects in MPro (31.79%), our identified TFPs displayed eight positive effects and two negative effects, in both cases very close to zero.

We also compared our approach against a comprehensive analysis of SARS-CoV-2 fitness effects that relies on contrasting the expected number of independent occurrences of a mutation in four-fold degenerate sites (absence of selection) against the actually observed appearances ^52^. Of the total of 539 TFPs, 80 had positive and 459 had negative effects. If considering the non-TFPs (351 and 3633, respectively), the odds ratio of a TFP having positive effect is 1.80 (95% CI: 1.37 to 2.35), which opposes to the DMS results. The distribution of positive (Extended Data Figure S13A) and negative (Extended Data Figure S13B) fitness effects over the entire SARS-CoV-2 genome is significantly different when comparing TFPs and non-TFPs (Wilcoxon signed-rank test p-values = 0.009 for positive and p<0.0001 for negative), with TFPs having less positive and less negative overall fitness (i.e. skewing towards zero) values (Extended Data Figure S13A-E). A possible explanation for this observation is that TFPs have moderate multilevel positive and negative selection effects. However, the compared method, which is based on fixed effects, would average out such effects making them approach zero because it only measures the magnitude of one level of selection.

## Discussion

We have presented a tree-based clustering method to investigatetransient selective forces acting on SARS-CoV-2 lineages and mutations through the calculation of three statistics (ratio of sizes, ratio of persistence time and logistic growth rates between sister clades) and extraction of clades containing values of those statistics below small cluster threshold cutoffs. To mitigate the inclusion of spurious sites, we included only recurring clade-defining mutations (homoplasies) across cluster thresholds with low associated FDRs and excluded probable sequencing artifacts. Our approach provides a scalable way to analyse huge genomic datasets with >1 million sequences for multilevel selection while also accounting for shared ancestry.

Although the COVID-19 pandemic offered the opportunity to collate genomic datasets of unprecedented sizes, estimating the transmission fitness of individual polymorphisms in this context is challenging. In the early epidemic, inference of sites under positive selection was hampered by low sensitivity given the small genetic diversity of the virus, probably resulting in underestimated estimates of fitness costs. For example, a phylogenetic approach was developed to quantify imbalance in clades containing recurrent mutations ^9^, and this approach found a lack of evidence for increased transmissibility from recurrent SARS-CoV-2 mutations. However, this approach only used ≈50,000 sequences up to July 2020 and did not considered persistence times and growth rates as measures of differential fitness across clades of the phylogeny. Following the emergence of VOCs exhibiting elevated substitution rates, other attributes such as convergent evolution, sparse sampling, and vaccine-elicited immunity appeared as relevant confounding factors. Most importantly, the detection of positive selection does not necessarily imply enhanced transmissibility, and the effects of individual mutations on this trait will typically be modest ^10^.

Although we carefully considered convergent evolution in our approach by aggregating summary statistics across multiple occurrences of the same mutation, the statistical power is reduced when there are only a few recurrences, as observed here for SARS-CoV-2. For example, this limits our ability to disentangle context-dependent selection effects of TFPs, i.e., when particular mutations are only detrimental at the between-host level if they appear in one major lineage and not others. However, we believe realistic population genetic simulations and data from other pathogens in which within-host evolution is the major source of molecular diversity (e.g. HIV-1) may provide a better understanding of our capacity to detect multilevel selection more rigorously. Sampling biases and changes in the immunological landscape of the human population were not directly addressed here and deserve future developments. Phylogenetic tree estimation and molecular dating on such large-scale datasets also represented enormous challenges in the early pandemic, due to exceedingly high turnaround times and inference uncertainty. However, novel elegant developments allowed the increasingly fast and accurate merging of lineage-specific phylogenies into bigger trees ^40^, placing new sequences into existing phylogenies using parsimony methods and efficient data structures ^39^, and estimating time-scaled trees using a differentiable graphs for efficient calculation ^34^. These advancements in the field of phylogenetics were essential for this work to be possible. 

Genetic diversity in infected individuals is governed by repeated cycles of within-host (e.g. replication and immune escape pressures) and between-host processes (e.g. transmission bottlenecks), with the outcome of selection at each level having an effect on the other ^53^. The rapid accumulation of mutations in individuals ^54, 55^ with long-lasting chronic SARS-CoV-2 infection is hypothesised to contribute to the emergence of variants such as Alpha and Omicron ^56^. Thus the interaction of within-host and between-host selective processes can occasionally have very large epidemic-level effects.

The inspection of the global and lineage distributions of highly frequent TFP-homoplasies confirmed that these mutations generally emerge independently in multiple lineages but remain quite rare, which is consistent with a simultaneous within-host advantage and between-host disadvantage. This systematic investigation also emphasises the scarcity of experimental studies to characterise the functional impact of mutations outside the spike protein.

Our approach identified modest differences in multilevel selection signals across two different epidemic phases, lineages and genomic regions in the UK. We hypothesised that transient selective forces would become stronger after high-levels of convalescent and vaccine-induced immunity have been reached, but our results do not support this hypothesis. Our observation of approximately constant levels of transient selection across waves driven by extremely distinct variants may in part be driven by long-duration chronic infections which occur at low frequency and provide greater opportunities for accelerated within-host evolution favouring immune evasion. On the other hand, a systematic evaluation of longitudinal samples from chronically infected patients using a range of molecular dating methods found a lack of evidence for such higher within-host as opposed to between-host rates. These findings suggest that intrahost evolutionary rates have been overestimated and other considerations such as prolonged viral shedding and relapsing viral load dynamic should also be considered for a more comprehensive understanding of SARS-CoV-2 within-host dynamics ^57^. Our data did not include clinical covariates that would allow us to investigate the association of chronic infection or duration of infection and the presence of TFPs.

Sequencing of chronically-infected patients throughout multiple time points of their long-lasting infection provided external validation of our observed patterns. Nucleotide substitution rates were around twice as fast during chronic infections when compared with the global SARS-CoV-2 evolutionary rate ^58^. Additionally, mutations identified in the top 100 most frequent TFP-homoplasies by our approach such as S:E484K ^58– 61^, S:T95I ^60, 61^, ORF8:Y73C ^59^, ORF8:L118V ^58^, ORF1ab:S944L ^58^, and ORF1ab:T1543I ^58^ also emergedafter days of chronic infection. Although usually associated with immune escape and increased ACE2 affinity, these recurrent mutations lack the capacity to enhance transmission ^60, 61^ as demonstrated by their low epidemic-level frequency after multiple independent occurrences. Additionally, distinct viral populations appear to be residing in different niches ( *e.g.* organs) of a patient’s body ^61^ and an impaired immune system selects for mutations that confer intra-host replication and persistence ( *e.g.* immune evasion) as opposed to general acute infections, in which mutations favouring inter-host transmission are a major target of selective pressures ^60^. Remarkably, another study which investigated 27 chronic infections showed that certain recurrent mutations arising in such persistently infected patients do not appear at the between-host level, also indicating a tradeoff between antibody evasion and transmissibility as a plausible explanation ^61^. More recently, analysis of a large UK community surveillance study also demonstrated that particular mutations, including ORF1ab:T1638I (NSP3) and T4311I (NSP10), are recurrent in persistent infections but mildly deleterious at the between-host level. Conversely, many other recurrent mutations appearing in persistent infections were shown to be advantageous at the between-host level ^62^. Importantly, since our approach is especially designed to detect beneficial mutations at the within-host level, we do not expect that it directly captures mutations that are, at the same time, favourable for between-host transmission.

However, the comparison of our results against DMS experiments ^47– 51^ showed that although most TFPs matched negative (detrimental) fitness effects, some level of short-lived positive selection seems plausible for a few sites. We also contrasted our results against comprehensive SARS-CoV-2 fitness estimates from a method that compares the anticipated number of independent mutations at four-fold degenerate sites (where no selection occurs) with the observed mutation frequencies ^52^. It is important to emphasise that such approach is quantifying the magnitude of one level of selection only, while our approach attempts to identify multilevel selection. In other words, a single parameter is not sufficient to describe a site under multiple levels of selection, and future development is needed on statistical models for inferring multilevel selection effects. Although the fitness effects of these two approaches are not directly comparable, we found that TFPs tend to have fitness effects approaching zero, which can be explained by the simplistic assumption of fixed (unchanged) effects that would average the actual slightly positive and negative multilevel fitness estimates. Despite distance-based clustering in HIV networks having been extensively used as a proxy for transmissibility, this approach is generally based on a cutoff from pairwise distances separating sequences ^21^. Consequently, poor specificity for variants negatively influencing fitness is evident (i) when a variant is isolated, occurring along a long branch not captured by distance threshold, (ii) when a variant is imported, and large genetic distances can reflect unsampled diversity in the country of origin or a rare recombination or hypermutation event, not necessarily reflecting a fitness cost. Our method addresses these limitations by incorporating the number of descendants, persistence times, and growth rates across sister clades with and without the mutations under investigation, and using independently-acquired substitutions to remove spurious relationships. Introducing these multiple sources of information can provide more accurate estimates, but also introduce biases. Primarily, our analysis is sensitive to sequencing artifacts. Although we used data from a highly standarised sequencing consortium (COG-UK), changes in primer sets after Omicron emergence ^42^, as well as sequencing coverage and base-calling errors can potentially influence our conclusions, as demonstrated by our several quality control and artifact removal methods employed. A second limitation is our focus on consensus sequences. By using our approach, some beneficial within-host mutations (TFPs) might be missed because minority variant transmission is rare in scenarios where the *de novo* mutation rate is low (i.e., mutation takes time to reach >50% frequency within a host) and the transmission bottleneck is tight, as observed in SARS-CoV-2 ^63^. A third caveat arises from the assumption of representative sampling. Although we utilised data from England during a period of proportional (to cases) community sampling to minimise this effect, the rate of sampling varied substantially over time and further analyses are needed to investigate the impact of non-representative sequencing in our approach. Moreover, sample density will certainly impact the sensitivity of the method to detect circulating TFPs. By definition, these variants will have low persistence and growth, and therefore will only be detectable when a sufficiently high sample density is achieved. A detailed quantitative understanding of detection thresholds will need to be carried out in future work. Lastly, the small absolute frequency of reoccurrence of some identified TFPs raises concerns about the false discovery rate of the proposed method. We believe that, given that comprehensive analyses of SARS-CoV-2 fitness effects ^52^ point to a nearly neutral between-host effect of synonymous mutations along the SARS-CoV-2 genome, our FDR estimates of ~10% (that uses TFP detection in synonymous sites as a proxy for false detection) is reasonable for these sites to be considered under putative multilevel selection.

## Conclusions

We developed a method designed to identify candidate sites under multilevel selection from >1.2 million SARS-CoV-2 sequences using rigorous quality control, statistical tests, and control for false detection. The comprehensive catalog of TFPs identified here and especially abundant in S, N, ORF3a, ORF7, and ORF8 suggest the existence of important tradeoffs between within-host replication and between-host transmission of SARS-CoV-2 that may warrant further experimental investigation and more realistic coalescent-based model developments.

## Data Availability

Zenodo: Underlying and Extended data for: Phylogenetic signatures reveal multilevel selection and fitness costs in SARS-CoV-2. https://doi.org/10.5281/zenodo.10276239 ^41^. This project contains the following underlying data: ExtendedData.pdf supplementary text, figures, and table cited in the paper ExtendedData_FileS1.txt - output of Poisson regressions across the ten employed cluster thresholds for the time period before Omicron BA.1.* emergence (early June 2020 to mid-November 2021) that tested whether TFPs were enriched in particular genomic regions or major PANGO lineages. ExtendedData_FileS2.txt - output of Poisson regressions across the ten employed cluster thresholds for the time period including Omicron BA.1.* emergence (early June 2020 to the end of April 2022) that tested whether TFPs were enriched in particular genomic regions or major PANGO lineages. ExtendedData_FileS3.zip - this ZIP file contains other four files: sc2_md_curated_WITH_Xs_Ns.rds - underlying preprocessed metadata file to use as input for mlscluster to reproduce the analysis. sc2_tre_curated.rds - underlying preprocessed time-scaled phylogenetic tree file to use in combination with the metadata file as input for mlscluster to reproduce the analysis. res_p2.rds - output of a time-consuming run of the mlsclust function ( https://github.com/mrc-ide/mlscluster/blob/main/R/mlsclust.R) for the period excluding Omicron (June 2020 to mid-November 2021). res_p3.rds - output of a time-consuming run of the mlsclust function for the period including Omicron (June 2020 to April 2022). Data are available under the terms of the Creative Commons Attribution 4.0 International license (CC-BY 4.0). Analysis code available from: https://github.com/vinibfranc/mlscluster-experiments Archived source code at time of publication: https://doi.org/10.5281/zenodo.10520059 ^31^ License: MIT

## Software availability

We implemented the inference methods described in this paper into a new R package entitled mlscluster Source code available from: https://github.com/ mrc-ide/mlscluster

Archived source code at time of publication: https://doi.org/10.5281/zenodo.10523291 ^29^

License: MIT
