# Supplementary material for: Phylogenetic signatures reveal multilevel selection and fitness costs in SARS-CoV-2
Source: Wellcome Open Res. 2024 Jul 24;9:85. Originally published 2024 Feb 19. [Version 2] doi: 10.12688/wellcomeopenres.20704.2 (PMC11316176; doi:10.12688/wellcomeopenres.20704.2)
Supplement: Peer review history

# Reviewer report: Reviewer response for version 2

**DOI:** 10.21956/wellcomeopenres.25140.r90710

**Published:** 2024-08-23

**Authors:** Mahan Ghafari

**Affiliations:** 1 University of Oxford, Oxford, UK

I thank the authors for their response and have no further comments.

Is the work clearly and accurately presented and does it cite the current literature?

Partly

If applicable, is the statistical analysis and its interpretation appropriate?

Yes

Are all the source data underlying the results available to ensure full reproducibility?

Yes

Is the study design appropriate and is the work technically sound?

Yes

Are the conclusions drawn adequately supported by the results?

Partly

Are sufficient details of methods and analysis provided to allow replication by others?

Yes

Reviewer Expertise:

Virus evolution, phylogenetics, and epidemiology.

I confirm that I have read this submission and believe that I have an appropriate level of expertise to confirm that it is of an acceptable scientific standard.

# Reviewer report: Reviewer response for version 2

**DOI:** 10.21956/wellcomeopenres.25140.r90711

**Published:** 2024-08-09

**Authors:** Ariane Weber

**Affiliations:** 1 Max Planck Institute of Geoanthropology, Jena, Thuringia, Germany

In my opinion, all previous comments are sensibly addressed in version two of the article and all questions answered thoroughly in the responses to the reviewer reports. I thank the authors for their careful consideration of the latter.

 I have no further comments to make.

Is the work clearly and accurately presented and does it cite the current literature?

Partly

If applicable, is the statistical analysis and its interpretation appropriate?

Yes

Are all the source data underlying the results available to ensure full reproducibility?

Yes

Is the study design appropriate and is the work technically sound?

Yes

Are the conclusions drawn adequately supported by the results?

Partly

Are sufficient details of methods and analysis provided to allow replication by others?

Yes

Reviewer Expertise:

Pathogen phylodynamics

I confirm that I have read this submission and believe that I have an appropriate level of expertise to confirm that it is of an acceptable scientific standard.

# Reviewer report: Reviewer response for version 1

**DOI:** 10.21956/wellcomeopenres.22912.r77882

**Published:** 2024-04-17

**Authors:** Ariane Weber

**Affiliations:** 1 Max Planck Institute of Geoanthropology, Jena, Thuringia, Germany

**Summary**

 In “Phylogenetic signatures reveal multilevel selection and fitness costs in SARS-CoV-2” Bonetti Franceschi and Volz present a tree-based method, *mlscluster*, to identify homoplasies that are associated with the most unbalanced parts of the respective tree and apply it to SARS-CoV-2 genome sequences sampled in England between June 2020 and April 2022. The method includes a Poisson regression model to test if the identified homoplasies are enriched in specific genome regions, between different time intervals or in major PANGO lineages.

**General opinion**

 The method represents a fast and scalable way of extracting patterns of transmission heterogeneity caused by individual mutations from phylogenetic trees. It is thereby of topical relevance, as exemplified by the empirical analysis included in the article. Although the authors scrutinise their results by the calculation of a False Discovery Rate, my main concern relates to the lack of a more thorough evaluation of the performance of the method (see major comments). I deem this of particular relevance with regards to the interpretation of the identified homoplasies as transmission fitness polymorphisms providing evidence of multilevel selection.

 All data necessary for and produced by the empirical analysis is publicly shared in a well-readable format, as well is all software. The article itself is sensibly structured and accompanied by visualisations for all important elements, *i.e. *methodology and results. In my opinion, the phrasing is lacking in clarity in some instances and would require rephrasing for easier readability (reason for response “Partly” to question “Is the work clearly and accurately presented and does it cite the current literature?”, see also minor comments). A more detailed discussion of the limitations of the method would improve the contextualisation, especially some, to me rather far-fetched, claims would need qualifying (see minor comments).

**Major comments**

1. To my understanding, the methodological result of this study is a list of mutations that have occurred multiple times in the phylogeny in clades that are characterised by relatively small values of one or all of the test statistics (size, persistence, growth). By arguing that the recurrence is evidence for a within-host transmission advantage and the clade characteristics for a between-host disadvantage, these homoplasies are interpreted as transmission fitness polymorphisms (TFP) and evidence for multilevel selection in SARS-CoV-2. However, especially given the small absolute frequency of reoccurrence of most identified TFPs, this interpretive step is not straightforward and a bridge between result and interpretation is missing. I would therefore recommend the addition of either a validation study on either synthetic or empirical data (providing evidence that the identified mutations are really the result of a transient transmission advantage) or of a very well-constructed argument supported by appropriate literature. (This is the reason why I chose the response “Partly” to “Are the conclusions drawn adequately supported by the results?”)
2. Which target node conditions were used for the SARS-CoV-2 tree? How are the choices justified and how sensitive are the results to changes in these parameters? I do see in the code that the default values are min_descendants = 10, max_descendants = 20*10^3, min_cluster_age_yrs = 1/12, min_date = max_date = NULL, but I cannot find information regarding it in the manuscript.
3. Why is caution only recommended starting from thresholds associated with an FDR of around 40%?
4. If sister clades both qualify as target nodes, is the same (but reversed) comparison done twice and counted in the empirical distribution?
5. Are some parts of the tree counted multiple times, *e.g.* if a parent and child node both qualify as target nodes with the same defining mutation?
6. As demographic factors will still influence the clade characteristics, even in the presence of multilevel selection, how many independent samples are necessary, *i.e.* how often would the mutations have to be observed in the tree, to draw statistically reliable conclusions (see also Supplementary Figure S17 in van Drop et al. 2020)? What is empirically observed for the reported homoplasies?
7. Do the identified homoplasies also appear in parts of the tree that are not characterised as imbalanced?
8. How are multiple defining mutations in one clade handled?
9. I would recommend expanding the Discussion in the following two points:
  - More thorough discussion of the limitations that are already listed (how does the method deal with the challenges described in the second paragraph of the Discussion) and additional ones that apply ( *e.g.*, phylogenetic uncertainty and reliable tree inference as potentially time-consuming preprocessing step, statistical power from only few recurrences)
  - Explanation for and discussion of the pronounced differences observed between the mlscluster and HyPhy results.
10. I would recommend a more careful phrasing of the possible explanation of the observed TFP-homplasy pattern by accelerated within-host rates, as I see no ground justifying a connection presented in the study and too few references showing evidence of the connection and rate acceleration (see also preprint [1].

**Minor comments**

1. Abstract:
  - “We applied this method **for** a SARS-CoV-2 time-calibrated phylogeny..” --> “We applied this method **to** a SARS-CoV-2 time-calibrated phylogeny..”
  - To my understanding, the conclusion statement is not supported by the study results (see also major comment 1), as they do not allow to draw direct conclusions about within-host vs. between-host evolution. I would recommend rephrasing this.
2. Plain Language Summary:
  - I would recommend using “clade” instead of “lineage” for consistency.
  - “…highlighting the existence of important tradeoffs in selection between intrahost replication and inter-host transmission”: I would recommend rephrasing this or providing evidence that the identified homplasies are necessarily the product of multilevel selection (see major comment 1)
3. Introduction:
  - A literature reference is missing for the statement “In molecular epidemiological studies, a set of particularly scalable approaches have been developed based on the calculation of phylogenetic clusters comprising two or more closely related samples.” Additionally, I would suggest replacing “calculation” with, for example, “identification”, “detection” or similar.
  - Either a literature reference or a clearer argument is missing for the statement “Furthermore, there is considerable scope to improve on distance-based genetic clustering methods because such approaches will potentially have poor specificity for variants that negatively influence fitness.”
  - In the sentence following the one cited in the previous point the reference to SARS-CoV-2 is duplicated.
  - A formal definition of “transmission fitness polymorphism” is missing.
  - “We demonstrated its applicability through the analysis of a representative >1.2 million SARS-CoV-2 genomic data set from England…” should, *e.g.*, rather be phrased as “We demonstrated its applicability through the analysis of a representative SARS-CoV-2 data set comprising >1.2 million genome sequences from England…”
  - “By providing a comprehensive catalog of the main sites driving multilevel selective pressures throughout the SARS-CoV-2 genome, we also expand the understanding of [ ***the***] SARS-CoV-2 fitness landscape outside the well-studied spike protein”. I would recommend rephrasing of this sentence, as (i) the identified sites are not the drivers of the selective pressures, rather the result of them and (ii) see major comment 1.
4. Methods
  - Usage of “node” and “clade” is inconsistent. I would recommend primarily using only one of the two terms and, if necessary, write “node” only to refer to the actual internal node and “clade” when referring to the whole subtree arising from said “node”.
  - “Assume two clades u (target clade) and v (comparator/sister) organised in a time-scaled tree t and sharing ancestry ( *i.e.* the same defining mutations).” --> “Assume two clades u (target clade) and v (comparator/sister) organised in a time-scaled tree t and sharing **full **ancestry ( *i.e.* the same defining mutations).”
  - “The persistence time (given by a in Figure 2, is defined as..” --> “The persistence time (given by a in Figure 2 **)**, is defined as..” (closing bracket at wrong position)
  - “…which can be their sister clade (the clade sharing an immediate ancestor assuming bifurcating phylogenetic relationship) or against all other clades…” --> “…which can be their sister clade (the clade sharing an immediate ancestor assuming bifurcating phylogenetic relationship) o **r a**ll other clades…”
  - Unclear if ‘clade size’ refers to S_uv or n_u
  - I would recommend explicitly stating that in this study it is assumed that a homplasy that arises in a subtree for which the statistics fall below a low quantile of the empirical distribution (see sentence “… for identifying especially convergently acquired mutations (homplasies) that are detrimental for transmission (within a low quantile of the probability distribution of at least one of the three statistics”).
  - The observed distribution of each of the three statistics in the tree is formally not a probability distribution. I would recommend replacing this terminology.
  - “Given this efficient way to visit nodes of the tree and edge lengths, we can easily extract the parameters of interest (e.g. the time of the most recent common ancestor of each node, …” --> “Given this efficient way to visit nodes of the tree and edg **e,** we can easily extract the parameters of interest (e.g. the time of the most recent common ancestor of each **clade**, …”
  - “Target nodes are extracted based on the following conditions …” The following needs rephrasing, as it currently reads as if only the node with the smallest number of descendants etc. is kept.
  - Definition of “sharing ancestry” as same defining mutations between sister clades and “defining mutation” in one sister clade is contradicting.
  - Which homoplasy annotations are used later in the text?
  - “We intended to make the method flexible by creating a parameter that specifies in how many percentiles the statistic should be splitted...”: Isn’t it rather a parameter that specifies which percentile to use (e.g. 2%)?
  - I find the terminology “cluster threshold” confusing, as the output of the method is not primarily a clustering of parts of the tree into groups, but rather the identification of recurring mutations based on relative differences between any sister clades (passing the filtering conditions).
  - A literature reference is missing for the ML tree inference.
  - “We tested our approach using two COVID-19 pandemic time-periods: (i) from June 01, 2020 (including Wuhan/WH04/2020 reference sequence as root of the phylogeny) to November 15, 2022 (before Omicron BA.1.* variant emergence)..” --> “We tested our approach using two COVID-19 pandemic time-periods: (i) from June 01, 2020 (including Wuhan/WH04/2020 reference sequence as root of the phylogeny) to November 15, **2021** (before Omicron BA.1.* variant emergence)..”
  - The sentence “For each period, 10 different thresholds (…) of the clustering statistics are computed” needs rephrasing, e.g. “For each period, 10 different thresholds (…) of the clustering statistics are considered”. I would also suggest adding “%” after each threshold value.
  - Alignment-aware artifact removal: Is it correct that this is only necessary if occurrences of “X” and “N” at each genome position are not randomly distributed over the tree?
  - Poisson regression: definition of i and j is missing
  - Definition of Y as TFP calls among the I polymorphic third codon position sites with >100 mutated sequences: Why 100?
  - “Multiplication by 100 transforms the probability of erroneously calling a TFP into a percentage for easier interpretation”: The FDR does not represent the probability of erroneously calling a TFP, I would recommend rephrasing this.
  - Closing bracket missing: “(see Methods: Statistical analysis for identifying genomic regions enriched for TFP **)**.”
  - “This independent analysis provides additional evidence that their evolution is consistent with a transient selective pressure.”: Why is this the case? To my understanding, the manual inspection of the relatively frequent TFP homoplasies mainly shows that the method worked as expected here (i.e. the identified homplasies are recurrent and in ‘small’ clades)
5. Discussion
  - “We have quantified transient selective forces acting on SARS-CoV-2 lineages and mutations…”: I would recommend rephrasing this, as the study results do not represent a quantification of the selective forces.
  - “After the emergence of VOCs with elevated substitution rates, other attributes…”: I would recommend rephrasing this, since it reads as if VOCs have elevated evolutionary rates.
  - “Genetic diversity in an infected individual is goverened by repeated cycles…” --> “Genetic diversity in infected individuals is goverened by repeated cycles…”
  - “Additionally, mutations identified in the top 100 most frequent TFP-homoplasies […] also emerged after days of chronic infection.”: Since they emerged within days, were they shown to only emerge in chronically infected individuals and not others?
6. Conclusions:
  - “We developed a method capable of identifying sites under multilevel selection...”: I would recommend being more careful with the wording here, see major comment 1.

Is the work clearly and accurately presented and does it cite the current literature?

Partly

If applicable, is the statistical analysis and its interpretation appropriate?

Yes

Are all the source data underlying the results available to ensure full reproducibility?

Yes

Is the study design appropriate and is the work technically sound?

Yes

Are the conclusions drawn adequately supported by the results?

Partly

Are sufficient details of methods and analysis provided to allow replication by others?

Yes

Reviewer Expertise:

Pathogen phylodynamics

I confirm that I have read this submission and believe that I have an appropriate level of expertise to confirm that it is of an acceptable scientific standard, however I have significant reservations, as outlined above.

## References

[1] : Phylogenetic meta-analysis of chronic SARS-CoV-2 infections in immunocompromised patients shows no evidence of elevated evolutionary rates. *bioRxiv* .2023; 10.1101/2023.11.01.565087 10.1101/2023.11.01.565087

# Response

**Published:** 2024-07-15

**Authors:** Vinicius Bonetti Franceschi

**Affiliations:** Infectious Disease Epidemiology, Imperial College London, London, England, UK

Thank you very much for your time to review the paper. We apologise for the delay in getting back to you. This was due to other concurring projects and our tentative to address the high number of comments raised by the three reviewers in the best possible way. We appreciate your very detailed and thoughtful comments, and believe that they helped to improve the readability and contextualisation of the paper. Most importantly, we tried to make the wording less definitive about the sites actually being TFPs and explain that these will be used as input for more rigorous coalescent-based modelling in the near future to confirm or not their multilevel selection effects.  Please see below the response to the specific points:

**Major comments**

**1. To my understanding, the methodological result of this study is a list of mutations that have occurred multiple times in the phylogeny in clades that are characterised by relatively small values of one or all of the test statistics (size, persistence, growth). By arguing that the recurrence is evidence for a within-host transmission advantage and the clade characteristics for a between-host disadvantage, these homoplasies are interpreted as transmission fitness polymorphisms (TFP) and evidence for multilevel selection in SARS-CoV-2. However, especially given the small absolute frequency of reoccurrence of most identified TFPs, this interpretive step is not straightforward and a bridge between result and interpretation is missing. I would therefore recommend the addition of either a validation study on either synthetic or empirical data (providing evidence that the identified mutations are really the result of a transient transmission advantage) or of a very well-constructed argument supported by appropriate literature. (This is the reason why I chose the response “Partly” to “Are the conclusions drawn adequately supported by the results?”)**

 Response: Thank you very much for your comment. We tried to make it clearer now throughout the paper that the objective of mlscluster is to detect CANDIDATE sites under multilevel selection to feed into more complex coalescent-based models that we are still developing. These models will account for and have at least two selection coefficients (within and between-host). Therefore, mlscluster narrows down our search by making us start from the sites that are more likely to be TFPs and not considering every single site in the genome, which would probably be prohibitive. We are currently working on such methods and simulations to compare the accuracy of mlscluster vs these coalescent-based models, and will present such benchmarks in future work. We agree that the small absolute frequency of reoccurrence of most identified TFPs makes the interpretation weaker. But given that comprehensive analyses of SARS-CoV-2 fitness effects (doi: 10.1093/ve/veae026) point to a nearly neutral effect of synonymous mutations along the SARS-CoV-2 genome, we believe that our FDR of ~10% (that uses TFP detection in synonymous sites as a proxy for false detection) for threshold = 2% is reasonable enough for these sites to be considered under putative multilevel selection. These, in turn, will need further coalescent-based modelling or experimental studies to confirm the existence of multilevel selection effects. We included such explanation at the end of the 'Discussion'. Location of change in manuscript: R3#1 (Abstract, Plain Language Summary, Introduction, Discussion, Conclusions)

2. Which target node conditions were used for the SARS-CoV-2 tree? How are the choices justified and how sensitive are the results to changes in these parameters? I do see in the code that the default values are min_descendants = 10, max_descendants = 20*10^3, min_cluster_age_yrs = 1/12, min_date = max_date = NULL, but I cannot find information regarding it in the manuscript.

 Response: The default values of (i) 10, (ii) 20×10^-3 , and (iii) 1/12 (1 month) were chosen to avoid ratios being taken from small or unreliable clade sizes/persistence times, an unrealistically high number of viral generations, and very short timeframes. These values were selected based on previous experience with UK SARS-COV-2 analyses using a similar software developed by our research group (doi: 10.1016/j.ebiom.2023.104939). Unfortunately, a systematic evaluation of how these parameters would influence the results was not performed, but is planned for future applications of this method. We have included such clarifications in the revised manuscript. Location of change in manuscript: R3#2

**3. Why is caution only recommended starting from thresholds associated with an FDR of around 40%?**

 Response: We agree that this statement was not rigorous enough and changed it accordingly to recommend caution mainly starting at threshold = 5% that has an FDR ~ 20%. Location of change in manuscript: R3#3

**4. If sister clades both qualify as target nodes, is the same (but reversed) comparison done twice and counted in the empirical distribution?**

 Response: Yes, reversed comparisons between sister clades are included in our empirical distribution without any controls to avoid counting them twice. While it is true that the ratios are reciprocals and a log-transformation could be used to make them symmetric, our objective is to aggregate transmission fitness for all target nodes (and their resulting defining mutations) against their sisters. This means we are interested in capturing all comparisons, regardless of whether they are reversed. Excluding one of the two ratios or transforming them would not align with our goal of aggregating of transmission fitness. Therefore, we include both ratios to ensure that all potential relationships are considered in our analysis.

**5. Are some parts of the tree counted multiple times, e.g. if a parent and child node both qualify as target nodes with the same defining mutation?**

 Response: A child clade will have the mutations of the parent and their own. We only consider a defining mutation if it does happen in the target clade in a percentage > 75% across its tips while occurring in < 75% of the tips in the sister clade. Therefore, since the target and sister will carry the mutations of their parent, extracting the difference will remove such mutations and only keep the ones that are really defining, therefore not counting the parent mutations over and over. One of the reasons we do the alignment-aware artifact removal is because some tips in a clade could not have some mutations found in their parent called due to lack of sequencing coverage at that position, and that could make us call mutations that are not actually defining multiple times.

**6. As demographic factors will still influence the clade characteristics, even in the presence of multilevel selection, how many independent samples are necessary, i.e. how often would the mutations have to be observed in the tree, to draw statistically reliable conclusions (see also Supplementary Figure S14 in van Drop et al. 2020)? What is empirically observed for the reported homoplasies?**

 Response: Sample density will certainly impact the sensitivity of the method to detect circulating TFPs. By definition, these variants will have low persistence and growth, and therefore will only be detectable when a sufficiently high sample density is achieved. A detailed (quantitative) understanding of detection thresholds will need to be carried out in future work, but in the current paper we have added some discussion of these issues. Location of change in manuscript: R3#6

**7. Do the identified homoplasies also appear in parts of the tree that are not characterised as imbalanced?**

 Response: Thank you for such a great question. Yes, I compared the frequencies of all non-synonymous homoplasies found in the tree (n=4692) against the detected TFP-homoplasies (cluster threshold=2%, n=543). This means that only 11.57% of all non-synonymous homoplasies are also detected as TFP-homoplasies. The median difference in frequency for homoplasies detected at both (as general homoplasy [i.e. non-TFP] and as TFP) is 4, mean=8.52, and IQR=13. Non-synonymous TFP-homoplasy median frequency is 2, mean=2.45, and IQR=0.5, while non-synonymous general homoplasy median frequency is 3, mean=6.73, and IQR=4. So this preliminary investigation suggests that the identified homoplasies also appear in parts of the tree not characterised as imbalanced (in this case above the 2% cluster threshold) roughly a median of 4 times, but the variation can be quite large. We will not include such results in the manuscript, but will think of ways of formally quantifying such differences in future work.

**8. How are multiple defining mutations in one clade handled?**

 Response: The defining mutations of each clade are computed and the associated statistics (ratio of sizes, ratio of persistence time, and logistic growth rate) of the clade are attached to them, regardless of the clade having none (polytomy), one or multiple defining mutations. In the final data.frame, the mutations are 'individualised' to find the ones that happen independently in different parts of the tree that fall below the specified cluster threshold.

**9. I would recommend expanding the Discussion in the following two points:** **9.1. More thorough discussion of the limitations that are already listed (how does the method deal with the challenges described in the second paragraph of the Discussion) and additional ones that apply (e.g., phylogenetic uncertainty and reliable tree inference as potentially time-consuming preprocessing step, statistical power from only few recurrences)**

 Response: Thank you very much for your recommendation. We expanded the existing discussion to address the limitations already listed, although sampling biases is more thoroughly discussed by the end of the discussion and changes in immunological landscape were not considered. The additional points were also incorporated into the discussion. Location of change in manuscript: R3#9.1

**9.2. Explanation for and discussion of the pronounced differences observed between the mlscluster and HyPhy results.**

 Response: Thank you. This was also pointed out by Reviewer#2. The minimal overlap suggests that these methods are capturing different features / selective pressures, as expected. We included this clarification in the 'results' section. Location of change in manuscript: R3#9.1

**10. I would recommend a more careful phrasing of the possible explanation of the observed TFP-homplasy pattern by accelerated within-host rates, as I see no ground justifying a connection presented in the study and too few references showing evidence of the connection and rate acceleration (see also preprint [1].**

 Response: Thank you very much for the recommendation. We agree this point deserves further discussion and a more careful phrasing, and included a statement in the discussion citing the suggested paper, which indeed does a great systematic investigation of the within-host evolutionary rates in chronically infected SARS-CoV-2 patients. Location of change in manuscript: R3#10

**Minor comments**

**Abstract:** **11.1. “We applied this method for a SARS-CoV-2 time-calibrated phylogeny..” --> “We applied this method to a SARS-CoV-2 time-calibrated phylogeny**..”

 Response: Fixed. Location of change in manuscript: R3#11.1

**11.2. To my understanding, the conclusion statement is not supported by the study results (see also major comment 1), as they do not allow to draw direct conclusions about within-host vs. between-host evolution. I would recommend rephrasing this.**

 Response: Thank you very much, we agree with the recommendation and rephrased it to make it clear that these sites are not mechanistically (using e.g. realistic simulations) proven to be under multilevel selection. Location of change in manuscript: R3#11.2 **Plain Language**

**Summary:** **12.1. I would recommend using “clade” instead of “lineage” for consistency.** 

 Response: Thank you for the suggestion. We agree using "clade" is better for consistency and adjusted accordingly. We also rephrased the following to avoid repetition and provide additional clarification: "growth rates of clades carrying a specific mutation in comparison with their immediate sisters without the mutation". Location of change in manuscript: R3#12.1

**12.2. “…highlighting the existence of important tradeoffs in selection between intrahost replication and inter-host transmission”: I would recommend rephrasing this or providing evidence that the identified homplasies are necessarily the product of multilevel selection (see major comment 1)**

 Response: Similar to 11.2. Location of change in manuscript: R3#12.2

**Introduction: 13.1. A literature reference is missing for the statement “In molecular epidemiological studies, a set of particularly scalable approaches have been developed based on the calculation of phylogenetic clusters comprising two or more closely related samples.” Additionally, I would suggest replacing “calculation” with, for example, “identification”, “detection” or similar.**

 Response: Thank you for noticing this. We included a reference to the most widely used distance-based method (HIV-TRACE) and to a paper that evaluates the limitations of this and other similar methods. We also replaced "calculation" with "detection". Location of change in manuscript: R3#13.1

**13.2. Either a literature reference or a clearer argument is missing for the statement “Furthermore, there is considerable scope to improve on distance-based genetic clustering methods because such approaches will potentially have poor specificity for variants that negatively influence fitness.”**

 Response: We included a clarification on why these approaches are expected to present poor specificity for variants that negatively influence fitness. They were demonstrated to be systematically biased to detect variation in sampling rates instead of transmission rates. Location of change in manuscript: R3#13.2

**13.3. In the sentence following the one cited in the previous point the reference to SARS-CoV-2 is duplicated.**

 Response: We are not sure we understand this point. Actually, in this sentence: "During the past few years, positive and negative selection in SARS-CoV-2 have mainly been investigated using methods that rely on synonymous rate variation across sites/branches (26, 27) , and results from these approaches on SARS-CoV-2 comprehensive datasets are available for comparison (28)" reference 27 refers to the HyPhy method description paper and reference 28 to one of its applications to SARS-CoV-2 by the same author.

**13.4. A formal definition of “transmission fitness polymorphism” is missing.**

 Response: We agree it was not defined clearly, so we included a definition in the 'introduction' section. Location of change in manuscript: R3#13.4

**13.5. “We demonstrated its applicability through the analysis of a representative >1.2 million SARS-CoV-2 genomic data set from England…” should, e.g., rather be phrased as “We demonstrated its applicability through the analysis of a representative SARS-CoV-2 data set comprising >1.2 million genome sequences from England…”**

 Response: This sentence has been changed accordingly. Location of change in manuscript: R3#13.5

**13.6. “By providing a comprehensive catalog of the main sites driving multilevel selective pressures throughout the SARS-CoV-2 genome, we also expand the understanding of [the] SARS-CoV-2 fitness landscape outside the well-studied spike protein”. I would recommend rephrasing of this sentence, as (i) the identified sites are not the drivers of the selective pressures, rather the result of them and (ii) see major comment 1.**

 Response: We have rephrased it as "potentially resulting from multilevel selective pressures". Location of change in manuscript: R3#13.6

**Methods: 14.1. Usage of “node” and “clade” is inconsistent. I would recommend primarily using only one of the two terms and, if necessary, write “node” only to refer to the actual internal node and “clade” when referring to the whole subtree arising from said “node”.**

 Response: We agree using only one of the terms avoids confusion, and therefore chose "clade". Then, all instances of "node" were replaced by "clade" and some minor edits were made to avoid repetition of "clade" too many times. Location of change in manuscript: R3#14.1 (in the first replacement) and throughout the manuscript

**14.2. “Assume two clades u (target clade) and v (comparator/sister) organised in a time-scaled tree t and sharing ancestry (i.e. the same defining mutations).” --> “Assume two clades u (target clade) and v (comparator/sister) organised in a time-scaled tree t and sharing full ancestry (i.e. the same defining mutations).”**

 Response: I have added "full" as suggested, and also added the clarification that the ancestry is up to the point they diverge and therefore present their own exclusive defining mutations. Location of change in manuscript: R3#14.2

**14.3. “The persistence time (given by a in Figure 2, is defined as..” --> “The persistence time (given by a in Figure 2), is defined as..” (closing bracket at wrong position)**

 Response: Fixed. Location of change in manuscript: R3#14.3

**14.4. “…which can be their sister clade (the clade sharing an immediate ancestor assuming bifurcating phylogenetic relationship) or against all other clades…” --> “…which can be their sister clade (the clade sharing an immediate ancestor assuming bifurcating phylogenetic relationship) or all other clades…”**

 Response: Fixed. Location of change in manuscript: R3#14.4

**14.5. Unclear if ‘clade size’ refers to S_uv or n_u**

 Response: Thanks for noticing this. It referred to n_u and n_v and not to the ratio S_uv. We tried to rephrase this to ensure the meaning is not dubious. Location of change in manuscript: R3#14.5

**14.6. I would recommend explicitly stating that in this study it is assumed that a homplasy that arises in a subtree for which the statistics fall below a low quantile of the empirical distribution (see sentence “… for identifying especially convergently acquired mutations (homplasies) that are detrimental for transmission (within a low quantile of the probability distribution of at least one of the three statistics”).**

 Response: We agree that the suggested change clarify the analytic methods used, making it easier to understand and technically more accurate. It is now incorporated into the manuscript. Location of change in manuscript: R3#14.6

**14.7. The observed distribution of each of the three statistics in the tree is formally not a probability distribution. I would recommend replacing this terminology.**

 Response: We agree and have fixed this throughout the manuscript. Location of change in manuscript: R3#14.7

**14.8. “Given this efficient way to visit nodes of the tree and edge lengths, we can easily extract the parameters of interest (e.g. the time of the most recent common ancestor of each node, …” --> “Given this efficient way to visit nodes of the tree and edge, we can easily extract the parameters of interest (e.g. the time of the most recent common ancestor of each clade, …”**

 Response: Fixed. Location of change in manuscript: R3#14.8

**14.9.“Target nodes are extracted based on the following conditions …” The following needs rephrasing, as it currently reads as if only the node with the smallest number of descendants etc. is kept.**

 Response: Thank you very much for noticing this. We have rephrased accordingly and believe the sentence implies the intended meaning now. Location of change in manuscript: R3#14.9 

**14.10. Definition of “sharing ancestry” as same defining mutations between sister clades and “defining mutation” in one sister clade is contradicting.**

 Response: We are not sure we completely understand this statement, but we believe this was accordingly addressed together with 14.2 above. The main idea is to compare clades carrying a specific defining mutation against their immediate sisters without the mutation. Location of change in manuscript: R3#14.10

**14.11. Which homoplasy annotations are used later in the text?**

 Response: Homoplasies are annotated in the text and figures using the standard protein:{ancestral_aminoacid}{aminoacid_coordinate}{mutated_aminoacid} notation to make them consistent and comparable with other studies. The specific annotations (e.g. regions of interest including RBD and NTD of spike) are mentioned when appropriate to illustrate important results, in table 1 ('Genomic region' column), and in the detailed CSV outputs of the package.

**14.12. “We intended to make the method flexible by creating a parameter that specifies in how many percentiles the statistic should be splitted...”: Isn’t it rather a parameter that specifies which percentile to use (e.g. 2%)?**

 Response: 'quantile_choice' parameter specifies in how many percentiles the statistics should be splitted. For example, 'quantile_choice=1/100' (default) splits from 1% to 100% in intervals of 1%, 1/400 splits from 0.25% to 100% in intervals of 0.25%. The later allows more strict "cluster thresholds" to be used (e.g. 0.25%). Each statistic has its own parameter to set the percentile to use ('quantile_threshold_ratio_sizes', 'quantile_threshold_ratio_persist_time', and 'quantile_threshold_logit_growth'). Again, this option just makes the method more flexible, but it is recommended to consider the same threshold for all three statistics (which was the only scenario we tested). These options are properly documented in the R package, available using: help("run_diff_thresholds").

**14.13. I find the terminology “cluster threshold” confusing, as the output of the method is not primarily a clustering of parts of the tree into groups, but rather the identification of recurring mutations based on relative differences between any sister clades (passing the filtering conditions).**

 Response: Thank you very much for raising this terminology opinion. We respect it and agree it is a bit confusing, but preferred to use this instead of more technical terms such as "quantile threshold", "empirical distribution threshold", etc. It was a topic of discussion during the paper preparation and both of us agreed "cluster threshold" would not be a perfect name but better than other terms we could think of.

**14.14. A literature reference is missing for the ML tree inference.**

 Response: Thank you for noticing this. The citation to FastTreeMP, UShER, and phylopipe is now incorporated into the revised manuscript. Location of change in manuscript: R3#14.14

14.15. “We tested our approach using two COVID-19 pandemic time-periods: (i) from June 01, 2020 (including Wuhan/WH04/2020 reference sequence as root of the phylogeny) to November 15, 2022 (before Omicron BA.1.* variant emergence)..” --> “We tested our approach using two COVID-19 pandemic time-periods: (i) from June 01, 2020 (including Wuhan/WH04/2020 reference sequence as root of the phylogeny) to November 15, 2021 (before Omicron BA.1.* variant emergence)..”

 Response: Fixed. Location of change in manuscript: R3#14.15

**14.16. The sentence “For each period, 10 different thresholds (…) of the clustering statistics are computed” needs rephrasing, e.g. “For each period, 10 different thresholds (…) of the clustering statistics are considered”. I would also suggest adding “%” after each threshold value.**

 Response: Fixed. Location of change in manuscript: R3#14.16

**14.17. Alignment-aware artifact removal: Is it correct that this is only necessary if occurrences of “X” and “N” at each genome position are not randomly distributed over the tree?**

 Response: We think it should be performed regardless of whether their occurrence is randomly distributed over the tree or not. Since the method relies on extracting homoplasies along the tree, it will be highly prone to artifacts as demonstrated by our Omicron case scenario, in which we found a massive enrichment for TFPs (due to the known Omicron sequencing dropout issues) when not performing such sanity checks.

**14.18. Poisson regression: definition of i and j is missing**

 Response: These are already defined. "(...) i polymorphic third codon position sites (...)" (count of polymorphic sites at third codon position) and "(...) j is the total number of polymorphic sites at first and second positions (...)". 

**14.19. Definition of Y as TFP calls among the I polymorphic third codon position sites with >100 mutated sequences: Why 100?**

 Response: This was selected after retrieving the number of polymorphic synonymous sites at each codon position for a couple of thresholds of mutated sequences (ranging from 10 to 1000). N=100 represents ~0.1% of the ~1.7 million sequences analysed, so a threshold as high as 1000 or 500 was excluding too many legitimate polymorphic sites and a threshold as low as 10 or 50 could include artifacts or spurious polymorphic sites. We included a summarised version of this explanation in the revised manuscript. Location of change in manuscript: R3#14.19

**14.20. “Multiplication by 100 transforms the probability of erroneously calling a TFP into a percentage for easier interpretation”: The FDR does not represent the probability of erroneously calling a TFP, I would recommend rephrasing this.**

 Response: Thanks for noticing this. You are correct. We rephrased it as "proportion of erroneous TFP calls". Location of change in manuscript: R3#14.20

**14.21. Closing bracket missing: “(see Methods: Statistical analysis for identifying genomic regions enriched for TFP).”**

 Response: Fixed. Location of change in manuscript: R3#14.21

**14.22. “This independent analysis provides additional evidence that their evolution is consistent with a transient selective pressure.”: Why is this the case? To my understanding, the manual inspection of the relatively frequent TFP homoplasies mainly shows that the method worked as expected here (i.e. the identified homplasies are recurrent and in ‘small’ clades)**

 Response: Thank you for the suggestion. We agree and rephrased it to highlight only that it shows that the method works as expected and provided additional explanation ("indicated by the recurrence of such mutations and their appearance in clades where the size, longevity or growth rate of the target clade is much smaller when compared to its sister(s).") Location of change in manuscript: R3#14.22

**Discussion:** **15.1. “We have quantified transient selective forces acting on SARS-CoV-2 lineages and mutations…”: I would recommend rephrasing this, as the study results do not represent a quantification of the selective forces.**

 Response: Agreed. We replaced "quantified" with "presented a tree-based clustering method to investigate". Location of change in manuscript: R3#15.1

**15.2. “After the emergence of VOCs with elevated substitution rates, other attributes…”: I would recommend rephrasing this, since it reads as if VOCs have elevated evolutionary rates.**

 Response: Thank you, we rephrased this for clarity. Location of change in manuscript: R3#15.2

**15.3. “Genetic diversity in an infected individual is goverened by repeated cycles…” --> “Genetic diversity in infected individuals is goverened by repeated cycles…**” 

 Response: Fixed. Location of change in manuscript: R3#15.3 

**15.4. “Additionally, mutations identified in the top 100 most frequent TFP-homoplasies […] also emerged after days of chronic infection.”: Since they emerged within days, were they shown to only emerge in chronically infected individuals and not others? ** Response: The studies cited investigated their occurrence specifically in chronically infected patients, and to the best of our knowledge these mutations were not demonstrated to be particularly prevalent at the population level (acute infections) in several lineages throughout the pandemic.

**Conclusions:** **16. “We developed a method capable of identifying sites under multilevel selection...”: I would recommend being more careful with the wording here, see major comment 1."**

 Response: Agreed. We replaced "capable" with "designed to identify candidate sites (...)". Location of change in manuscript: R3#16

# Reviewer report: Reviewer response for version 1

**DOI:** 10.21956/wellcomeopenres.22912.r76365

**Published:** 2024-04-11

**Authors:** Mahan Ghafari

**Affiliations:** 1 University of Oxford, Oxford, UK

Franceschi and Volz have developed a method for identifying mutations that have a transient selective advantage at the within-host level but are disadvantageous at the between-host level. Their method utilises three statistics based on the size, persistence time, and logistic growth rates of clades harbouring the target mutations. Their analysis of SARS-CoV-2 is an interesting one and their methodology appears sound. One external validation of their method is the identification of recurrent mutations found during chronic infections, including mutations outside Spike. I have a few comments:

**Precedence:**

 The authors' claim of being the first to identify mutations with complex fitness trade-offs may benefit from further clarification and context regarding previous research in this area. However, I think their approach to identifying such mutations is particularly useful for large-scale datasets. For instance, the work by Harari et al., which the authors also cited, found trade-offs between immune evasion (and viral replication) and transmissibility. They showed that certain recurrent within-host mutations in chronic infections do not appear at the between-host level, pointing to the trade-off between immune escape and transmissibility as the possible explanation.

 More recently, Ghafari et al. [1] demonstrated that certain mutations, such as T1638I in ORF1ab, are recurrent in persistent infections but are mildly deleterious at the between-host level, while many other recurrent mutations in persistent infections are also highly advantageous at the between-host level.

**Methods:**

 The assumption that artifacts ("X") would necessarily follow the majority allele at a given position is not immediately clear to me. Could the authors clarify what this assumption is based on or verify from base frequency files whether "X" is indeed the majority nucleotide in at least some cases?

 A potential limitation of this approach is its focus on consensus sequences. A de novo mutation reaching over 50% frequency within a host takes time, and given the tight transmission bottleneck, minority variant transmission is unlikely. Thus, at least some beneficial within-host mutations might be missed.

 It would be interesting to know if the authors have explored context-dependent selective advantage/disadvantage of TFPs, for example, whether certain mutations are only detrimental at the between-host level if they appear in one major lineage and not others.

**Results:**

 It is still unclear to me whether the set cluster thresholds chosen for the analyses correspond to all three considered statistics, some of them, or at least one.

 The significance of observing B.1.1.7 and AY.4.* enriched for TFP-homoplasies is not well explained. Should this be interpreted as these lineages being more likely to give rise to beneficial within-host mutations that are deleterious at the between-host level? If so, the biological explanation for such lineage-dependent effects needs some clarification.

 Given that the study period extends only until mid-2022, it would be worth clarifying whether any second-generation BA.2 lineages, as well as BA.5 or XBB, were included in the analysis or not for interested readers.

 The discordance between sites identified as under positive selection using mlscluster and the HyPhy-based approach is noteworthy. The minimal overlap suggests these methods are capturing different features. Some comments/clarification helps here.

**Discussion:**

 Bloom and Neher's [2] recent investigation into the between-host fitness effect of SARS-CoV-2 mutations across all major lineages pre- and post-Omicron uses the number of independent appearances of a mutation on a global phylogeny as a measure of mutation fitness effects (not cluster size or their persistence times). Their approach also seems to work particularly well in capturing deleterious or nearly neutral mutations at the between-host level. I would be curious to see some discussion on how the methods compare with the author's approach in this paper.

 The discussion on how this approach could be used to investigate the association between chronic infection, duration of infection, and the presence of TFPs is an interesting one. Given Ghafari et al.'s [1] findings that many mutations during persistent infections are also beneficial at the between-host level, would the authors expect that their approach cannot capture mutations that are both beneficial at the within- and between-host level?

 The authors have explained other caveats and advantages of their approach well in the context of existing literature.  

**Minor Points:**

 - TFP-homoplasies should be defined before being mentioned in the abstract.

 - The terms Upper and Lower Tier Local Areas need definitions before their abbreviations are used.

 - Consider changing "… up to July 2020 and has not considered persistence times" to "did not consider persistence times".

 - There seems to be an accidental text break in the following paragraph "… ORF1ab:T1543I also emerged after days of chronic infection" which seems unnecessary.

 - On figure 4, it would help to indicate which mutations belong to the intersection category (yellow). Are these S484 and S98?

 - The sentence "…it shows that the impact of very few mutations outside the S protein have been characterised experimentally" could be rephrased for clarity. The results highlight potential functionally important mutations outside the Spike protein that warrant further investigation rather than directly showing lack of experimental characterisation.

Is the work clearly and accurately presented and does it cite the current literature?

Partly

If applicable, is the statistical analysis and its interpretation appropriate?

Yes

Are all the source data underlying the results available to ensure full reproducibility?

Yes

Is the study design appropriate and is the work technically sound?

Yes

Are the conclusions drawn adequately supported by the results?

Partly

Are sufficient details of methods and analysis provided to allow replication by others?

Yes

Reviewer Expertise:

Virus evolution, phylogenetics, and epidemiology.

I confirm that I have read this submission and believe that I have an appropriate level of expertise to confirm that it is of an acceptable scientific standard, however I have significant reservations, as outlined above.

## References

[1] : Prevalence of persistent SARS-CoV-2 in a large community surveillance study. *Nature* .2024;626(8001) : 10.1038/s41586-024-07029-4 1094-1101 10.1038/s41586-024-07029-4 38383783 PMC10901734

[2] : Fitness effects of mutations to SARS-CoV-2 proteins. *Virus Evol* .2023;9(2) : 10.1093/ve/vead055 vead055 10.1093/ve/vead055 37727875 PMC10506532

# Response

**Published:** 2024-07-15

**Authors:** Vinicius Bonetti Franceschi

**Affiliations:** Infectious Disease Epidemiology, Imperial College London, London, England, UK

Thank you very much for your time to review the paper. We apologise for the delay in getting back to you. This was due to other concurring projects and our tentative to address the high number of comments raised by the three reviewers in the best possible way. We appreciate your detailed and interesting comments, and believe that they helped to improve the contextualisation of the paper.  Please see below the response to the specific points:

**Precedence:**

**1. The authors' claim of being the first to identify mutations with complex fitness trade-offs may benefit from further clarification and context regarding previous research in this area. However, I think their approach to identifying such mutations is particularly useful for large-scale datasets. For instance, the work by Harari et al., which the authors also cited, found trade-offs between immune evasion (and viral replication) and transmissibility. They showed that certain recurrent within-host mutations in chronic infections do not appear at the between-host level, pointing to the trade-off between immune escape and transmissibility as the possible explanation.**

 Response: Thank you very much for your comment. We agree the first version of the paper did an inadequate job of putting our results in the appropriate context of existing work. We also included the suggested discussion of the work by Harari that corroborates our findings. Location of change in manuscript: R2#1

**2. More recently, Ghafari et al. [1] demonstrated that certain mutations, such as T1638I in ORF1ab, are recurrent in persistent infections but are mildly deleterious at the between-host level, while many other recurrent mutations in persistent infections are also highly advantageous at the between-host level.**

 Response: Thank you very much for suggesting this reference. We included a statement in the 'discussion' citing such mutations that appear to be deleterious at the between-host level and that the majority of recurrent mutations in persistent infections you found in this large community study tend to be beneficial at the between-host level. Location of change in manuscript: R2#2

**Methods:**

**3. The assumption that artifacts ("X") would necessarily follow the majority allele at a given position is not immediately clear to me. Could the authors clarify what this assumption is based on or verify from base frequency files whether "X" is indeed the majority nucleotide in at least some cases? A potential limitation of this approach is its focus on consensus sequences. A de novo mutation reaching over 50% frequency within a host takes time, and given the tight transmission bottleneck, minority variant transmission is unlikely. Thus, at least some beneficial within-host mutations might be missed.**

 Response: Thank you for pointing this out. We understand it was probably not accurately described before. What we actually did was to compute the proportion of the most frequent mutation at a given site across all sequences within the clade and add up the frequency of the "X" or "N" (undetermined bases) at that site (across all sequences in that same clade). This assumption is based on the complete shared ancestry across sequences in that clade. In cases where "X" and "N" are the most frequently mutated characters at the given clade, we discard them to avoid the inclusion of artifacts. We added such clarification in 'Methods: Statistical analysis for identifying genomic regions enriched for TFPs ' and hope this is clear now. We agree the focus on consensus sequences brings limitations such as the one cited, so we included it in the 'discussion' with an appropriate reference. Location of change in manuscript: R2#3 (methods and discussion)

**4. It would be interesting to know if the authors have explored context-dependent selective advantage/disadvantage of TFPs, for example, whether certain mutations are only detrimental at the between-host level if they appear in one major lineage and not others.**

 Response: Thank you very much for such an interesting question. As pointed out by Reviewer 3, the statistical power to detect such relationships would be very limited given the lower frequency of TFP-homoplasies. We have not investigated these context-dependent relationships in depth because we think any conclusions from that would be highly speculative given these limitations. We included a statement in the 'discussion' (paragraph 3) mentioning this. We found some TFPs to occur in more than one major lineage and others to happen only in one, so probably the answer is yes, there are lineage-specific differences. Hopefully, analysis of genomic data from other viruses where multilevel selection is more common and some realistic coalescent-based simulations we are currently working on will help us to better understand these relationships. Location of change in manuscript: R2#4

**Results:**

**5. It is still unclear to me whether the set cluster thresholds chosen for the analyses correspond to all three considered statistics, some of them, or at least one.**

 Response: We agree this was not very clear. We incorporated this sentence: "homoplasies occurring in a subtree for which at least one of the three statistics fall below a low quantile [e. g., 2%] of the empirical distribution" into the 'Methods: Tree-based clustering algorithm implementation' section to clarify that the analysis is performed for homoplasies and associated clades detected by at least one of the three statistics. Location of change in manuscript: R2#5

6. The significance of observing B.1.1.7 and AY.4.* enriched for TFP-homoplasies is not well explained. Should this be interpreted as these lineages being more likely to give rise to beneficial within-host mutations that are deleterious at the between-host level? If so, the biological explanation for such lineage-dependent effects needs some clarification.

 Response: We do not intend to make any claims about the biological propensity of these variants to generate TFPs, but merely to make the observation which may generate new hypotheses. It is possible that this observation is driven by surveillance effects, since there was relatively deep sampling of B.1.1.7 and AY.4 that may have enabled the detection of more candidate TFPs. Location of change in manuscript: R2#6 

**7. Given that the study period extends only until mid-2022, it would be worth clarifying whether any second-generation BA.2 lineages, as well as BA.5 or XBB, were included in the analysis or not for interested readers.**

 Response: We agree it is important to emphasise what lineages were considered. Unfortunately, after Pillar 2 termination in the UK (around April 2022), sampling was quite biased towards hospitalised cases. It is important to have a well-defined sampling frame over the duration of the study, so we chose not to consider sequences taken after this time (April 2022). We included a statement in the beginning of 'results' to clarify that such lineages (second-generation BA.2, BA.5, XBB, etc) were not included in the analysis. Location of change in manuscript: R2#7 

**8. The discordance between sites identified as under positive selection using mlscluster and the HyPhy-based approach is noteworthy. The minimal overlap suggests these methods are capturing different features. Some comments/clarification helps here.**

 Response: Thank you very much for pointing this out. We agree it is important to explicitly say that this minimal overlap suggests that these methods are capturing different selective pressures. We included this clarification in the 'results' section. Location of change in manuscript: R2#8 

**Discussion:**

**9. Bloom and Neher's [2] recent investigation into the between-host fitness effect of SARS-CoV-2 mutations across all major lineages pre- and post-Omicron uses the number of independent appearances of a mutation on a global phylogeny as a measure of mutation fitness effects (not cluster size or their persistence times). Their approach also seems to work particularly well in capturing deleterious or nearly neutral mutations at the between-host level. I would be curious to see some discussion on how the methods compare with the author's approach in this paper.**

 Response: Thank you very much for the interest in such comparison. As also suggested by Reviewer#1, we added the comparison of our identified TFPs against the fitness effects estimated by Bloom & Neher and some discussion on how the methods compare and why they would lead to different results. Location of change in manuscript: R2#9 (Methods, Results, and Discussion) 

**10. The discussion on how this approach could be used to investigate the association between chronic infection, duration of infection, and the presence of TFPs is an interesting one. Given Ghafari et al.'s [1] findings that many mutations during persistent infections are also beneficial at the between-host level, would the authors expect that their approach cannot capture mutations that are both beneficial at the within- and between-host level?**

 Response: Since our approach is especially designed to detect beneficial mutations at the within-host level, we do not expect that it directly captures mutations that are, at the same time, favourable for between-host replication. We included such statement in the discussion. Location of change in manuscript: R2#10 

**11. The authors have explained other caveats and advantages of their approach well in the context of existing literature.**

 Response: Thank you for your positive feedback!

**Minor:**

**12. TFP-homoplasies should be defined before being mentioned in the abstract.**

 Response: Thank you very much for noticing this. It is fixed. Location of change in manuscript: R2#12

**13. The terms Upper and Lower Tier Local Areas need definitions before their abbreviations are used.**

 Response: Thank you very much. It is addressed. Location of change in manuscript: R2#13 

**14. Consider changing "… up to July 2020 and has not considered persistence times" to "did not consider persistence times".**

 Response: Fixed. Location of change in manuscript: R2#14

**15. There seems to be an accidental text break in the following paragraph "… ORF1ab:T1543I also emerged after days of chronic infection" which seems unnecessary.**

 Response: Thank you very much for pointing this out. We have tried to fix it but there still seems to be a formatting issue there, so left a comment for the editorial team. Location of change in manuscript: R2#15 

**16. On figure 4, it would help to indicate which mutations belong to the intersection category (yellow). Are these S484 and S98?**

 Response: These are S:A67V and S:E484K. Unfortunately, the available HyPhy analysis only contained the identified sites and not the actual replacements under positive selection. Consequently, the actual mutations and further annotations are presented in Table 1. We added this clarification in the legend of Figure 4. We believe the yellow color is sufficient to distinguish these sites in the 'intersection' category and these sites are mentioned in the 'TFPs along the SARS-CoV-2 genome and low concordance'. Location of change in manuscript: R2#16

**17. The sentence "…it shows that the impact of very few mutations outside the S protein have been characterised experimentally" could be rephrased for clarity. The results highlight potential functionally important mutations outside the Spike protein that warrant further investigation rather than directly showing lack of experimental characterisation.**

 Response: Thank you for your suggestion. We have rephrased the sentence for clarity. We believe this revision more accurately conveys the intended meaning. Location of change in manuscript: R2#17

# Reviewer report: Reviewer response for version 1

**DOI:** 10.21956/wellcomeopenres.22912.r76372

**Published:** 2024-03-28

**Authors:** Richard Neher

**Affiliations:** 1 University of Basel, Basel, Switzerland

Franchesci and Volz present a method to investigate the effects of specific mutations on viral fitness by comparing clades that carry a specific mutation to their immediate siblings without the mutation. The method relies on comparing the total size, the 'longevity', and the relative growth of the clades.

 The paper is rather hard to read. It is, for example, not said explicitly that the method is specifically looking at homoplasies and aggregating information across many occurrences of the same mutation. Instead the discussion is kept general (target/comparator) when it would help a lot if the canonical scenario was explained. There are several other ways in which the readability of this paper could be improved.

 The specific claim that the method allows to identify multi-level selection as opposed to simply aggregate transmission fitness is not well supported.

 Specific points:

 Introduction:

 - the first paragraph is oddly neutralist. For SARS-CoV-2 or the HA segment of influenza A, adaptation is common and we witness a repeated pattern of variant replacement. While it might still be technically true that the 'majority' of polymorphisms is neutral or weakly selected, this introduction strikes me as strange. Furthermore, discussing transient fitness advantage as an exception to the neutral majority is particularly weird given the rest of the article is about SC2 with frequent replacements driven by adaptive evolution. A useful reference in this context could be Kistler and Bedford [1]

 - The motivation with multi-level selection in HIV is a bit strange and does not have much to do with what is discussed in the manuscript. It is unclear that different within/between host evolutionary rates in HIV are directly related to multi-level selection through preferential transmission of ancestral variants, or whether they are the result of frequent reversions of host-specific adaptations, for example T-cell escape. Influenza virus would be a much better model for the SC2 analysis done here than HIV. Strelkowa and Laessig [2] might be useful context.

 - the 2nd and 3rd paragraph of the introduction are rather cryptic.

 - the idea of fitness inference from the shape of phylogenies was introduced much earlier, for example [3]

 - the most comprehensive estimates of (mostly deleterious) fitness effects of mutations in SC2 to date were probably published in [4]

 Methods:

 - throughout the manuscript, it remains unclear whether identification of sites with fitness effects is the primary result, or enrichment of such sites in major variants or regions. This makes the manuscript hard to follow.

 Results

 - the paragraphs in the second column of page 9 summarize counts and observations without putting them into context and it is unclear what the reader is supposed to take away from this discussion.

 - I believe the functional significance of ORF7 and 8 is still not very well understood.

 Discussion:

 - it remains unclear to me why the approach presented here specifically reveals 'multi-level selection'. The approach picks up signals of clade growth and thus aggregate transmission rate in the population. The fact that most mutations are rare isn't evidence for a within-host/between-host trade-off. This is a natural consequence of a very large densely sampled pathogen with rapid exponentially growing outbreaks.

 - the results would be more convincing if they would be quantitatively compared to inferences from other analyses or deep mutational scanning data.

Is the work clearly and accurately presented and does it cite the current literature?

No

If applicable, is the statistical analysis and its interpretation appropriate?

Yes

Are all the source data underlying the results available to ensure full reproducibility?

Yes

Is the study design appropriate and is the work technically sound?

Yes

Are the conclusions drawn adequately supported by the results?

Partly

Are sufficient details of methods and analysis provided to allow replication by others?

Yes

Reviewer Expertise:

Viral evolution.

I confirm that I have read this submission and believe that I have an appropriate level of expertise to confirm that it is of an acceptable scientific standard, however I have significant reservations, as outlined above.

## References

[1] : An atlas of continuous adaptive evolution in endemic human viruses. *Cell Host Microbe* .2023;31(11) : 10.1016/j.chom.2023.09.012 1898-1909.e3 10.1016/j.chom.2023.09.012 37883977 PMC12129310

[2] : Clonal interference in the evolution of influenza. *Genetics* .2012;192(2) : 10.1534/genetics.112.143396 671-82 10.1534/genetics.112.143396 22851649 PMC3454888

[3] : Predicting evolution from the shape of genealogical trees. *Elife* .2014;3: 10.7554/eLife.03568 10.7554/eLife.03568 25385532 PMC4227306

[4] : Fitness effects of mutations to SARS-CoV-2 proteins. *Virus Evol* .2023;9(2) : 10.1093/ve/vead055 vead055 10.1093/ve/vead055 37727875 PMC10506532

# Response

**Published:** 2024-07-15

**Authors:** Vinicius Bonetti Franceschi

**Affiliations:** Infectious Disease Epidemiology, Imperial College London, London, England, UK

Thank you very much for your time to review the paper. We apologise for the delay in getting back to you. This was due to other concurring projects and our tentative to address the high number of comments raised by the three reviewers in the best possible way. Thank you especially for suggesting the comparison against DMS and computational fitness effects. We believe such comparisons made our discussion richer, especially by pointing out how the methods are different and why they lead to different results. Most importantly, we tried to make the wording less definitive about the sites actually being TFPs and explain that these will be used as input for more rigorous coalescent-based modelling in the near future to confirm/disconfirm their multilevel selection effects. Please see below the response to the specific points:

**1. Franceschi and Volz present a method to investigate the effects of specific mutations on viral fitness by comparing clades that carry a specific mutation to their immediate siblings without the mutation. The method relies on comparing the total size, the 'longevity', and the relative growth of the clades.**

 Response: Thank you for this suggestion. We agree that this is indeed a good way of summarising the method. We incorporated "clades carrying a specific mutation in comparison with their immediate sisters without the mutation" into the "Plain Language Summary" to make it clearer. Location of change in manuscript: R1#1

**2. The paper is rather hard to read. It is, for example, not said explicitly that the method is specifically looking at homoplasies and aggregating information across many occurrences of the same mutation. Instead the discussion is kept general (target/comparator) when it would help a lot if the canonical scenario was explained. There are several other ways in which the readability of this paper could be improved. ** Response: We appreciate your comment. We made it explicit now in the 'Introduction' that the method is looking at homoplasies and aggregating summary statistics across many occurrences of the same mutation and tried to explain the canonical scenario further. As it was not pointed out by the other reviewers as a major issue, we will not perform additional major changes to improve the readability of the paper, but of course will take this suggestion into consideration for future work. Location of change in manuscript: R1#2 (Introduction and Methods: Tree-based clustering algorithm implementation)

**3. The specific claim that the method allows to identify multi-level selection as opposed to simply aggregate transmission fitness is not well supported.**

 Response: See point #12 for a more detailed response (as both comments 3 and 12 are quite similar).

**Introduction:**

**4. The first paragraph is oddly neutralist. For SARS-CoV-2 or the HA segment of influenza A, adaptation is common and we witness a repeated pattern of variant replacement. While it might still be technically true that the 'majority' of polymorphisms is neutral or weakly selected, this introduction strikes me as strange. Furthermore, discussing transient fitness advantage as an exception to the neutral majority is particularly weird given the rest of the article is about SC2 with frequent replacements driven by adaptive evolution. A useful reference in this context could be Kistler and Bedford [1]**

 Response: There is of course a broad spectrum of selective effects and it was not our intention to imply that all variation is neutral, although it is certainly the case that the distribution of selective effects observed in circulating virus is concentrated strongly around zero. We have revised the discussion to be more balanced. Location of change in manuscript: R1#4 

**5. The motivation with multi-level selection in HIV is a bit strange and does not have much to do with what is discussed in the manuscript. It is unclear that different within/between host evolutionary rates in HIV are directly related to multi-level selection through preferential transmission of ancestral variants, or whether they are the result of frequent reversions of host-specific adaptations, for example T-cell escape. Influenza virus would be a much better model for the SC2 analysis done here than HIV. Strelkowa and Laessig [2] might be useful context.**

 Response: We agree HIV-1 evolution is quite diverse when compared to SARS-CoV-2 or Influenza, but would like to keep HIV-1 as the most extreme (and probably best) example of documented multilevel selection. We are well aware that diverse mechanisms (variable immune selection, store-and-retrieve etc.) can generate recurrent evolutionary patterns in HIV-1, and HIV-1 is nevertheless a source of good examples of the effects we are looking for. We incorporated the potential mechanisms of HIV-1 multilevel selection as mentioned and appropriate references. We also included Influenza as the example of a virus that evolves more similarly to SARS-CoV-2 as suggested. Location of change in manuscript: R1#5

**6. The 2nd and 3rd paragraph of the introduction are rather cryptic.**

 Response: We tried to improve the readability of these two paragraphs, and hope it is easier to follow now. Location of change in manuscript: R1#6

**7. The idea of fitness inference from the shape of phylogenies was introduced much earlier, for example [3]**

 Response: Thank you very much for pointing this out. We were aware of the method, and it has been influential to our thinking, and we cited this paper in the revised version of the manuscript. Note however that the design and objectives of [3] are very different than our proposed method for identifying multi-level selection effects. Location of change in manuscript: R1#7

**8. The most comprehensive estimates of (mostly deleterious) fitness effects of mutations in SC2 to date were probably published in [4]**

 Response: Thank you very much for bringing this to our attention. Note, however, that the objective of our analysis is somewhat different than those carried out by [4] and others. We removed the sentence that our paper was the first to do such an analysis and performed comparison against the other suggested methods (see especially points #12 and #13 for more details). Location of change in manuscript: R1#8

**Methods:**

**9. Throughout the manuscript, it remains unclear whether identification of sites with fitness effects is the primary result, or enrichment of such sites in major variants or regions. This makes the manuscript hard to follow."**

 Response: Thank you very much for the feedback. The primary purpose of the paper is to characterise sites with potential multilevel fitness effects, but the analysis of enrichment in major lineages and genomic regions comes together to put those findings into context (i.e. the findings would not be realistic if not considering the context of lineage and genomic region the TFPs appear). So basically these findings are both quite important. The results section is separated in 'Lineages and genomic regions enriched with SARS-CoV-2 TFP-homoplasies' and ' TFPs along the SARS-CoV-2 genome and low concordance with positively selected sites' to provide this didactic separation among these result 'categories'.

**Results**

**10. The paragraphs in the second column of page 9 summarize counts and observations without putting them into context and it is unclear what the reader is supposed to take away from this discussion.**

 Response: Thank you for the feedback. We agree some of the paragraphs needed contextualisation and added them when relevant (e.g. explanation of HyPhy minimal overlap, adding counts normalised per site instead of absolute counts, clearly stating genomic regions and major lineages in top-ranked TFP-homoplasies). Location of change in manuscript: R1#10

**11. I believe the functional significance of ORF7 and 8 is still not very well understood. ** Response: We agree and therefore rephrased the parts of the manuscript that made such a statement about ORF7a and ORF8 (Introduction and results section). Location of change in manuscript: R1#11

**Discussion:**

**12. It remains unclear to me why the approach presented here specifically reveals 'multi-level selection'. The approach picks up signals of clade growth and thus aggregate transmission rate in the population. The fact that most mutations are rare isn't evidence for a within-host/between-host trade-off. This is a natural consequence of a very large densely sampled pathogen with rapid exponentially growing outbreaks. ** Response: There seems to be a misunderstanding that the proposed methodology would only identify rare polymorphisms, the frequency of which is, of course, very sensitive to population dynamics. On the contrary, the scanning methodology is intended to shortlist all mutations that meet several criteria (recurrence, low persistence, low logistic growth). Population dynamics ("rapid exponentially growing outbreaks") is certainly an inadequate explanation for a variant meeting all three criteria. We tried to make it clearer now throughout the paper that the objective of mlscluster is to detect CANDIDATE sites under multilevel selection to feed into more complex coalescent-based models that we are still working on. These models will account for and have at least two selection coefficients (within and between-host). Therefore, mlscluster narrows down our search by making us start from the sites that are more likely to be TFPs and not considering every single site in the genome, which would probably be prohibitive. We believe that by considering homoplasies (independent occurences of a mutation) in a subtree for which at least one of the three clade growth-derived statistics (ratio of sizes, ratio of persistence time, and logistic growth rate) fall below a low quantile (2%) of the empirical distribution, we are not just aggregating transmission rate in the population, but picking up these candidate TFPs that are detrimental to transmission because they lead, multiple times, to fewer offspring, shorter lifespan, or smaller growth rate. Note as well that we have characterised false discovery rates using 3rd codon position, and it is unlikely given the ~10% FDR that most of these outcomes are observed by chance. We believe that a greater certainty will be reached when we provide quantitative estimates of multilevel selection using more rigorous coalescent-based modelling in the near future. Location of change in manuscript: R1#12 (Abstract, Plain Language Summary, Introduction, Discussion, Conclusions)

**13. The results would be more convincing if they would be quantitatively compared to inferences from other analyses or deep mutational scanning data.**

 Response: Thank you very much for the suggestion, although please note that these methods are estimating fundamentally different quantities. We compared our approach against DMS results and comprehensive Bloom & Neher fitness estimates (from point 8). Our main conclusion is that the sites we identified under multilevel selection (TFPs) tend to have fitness effects that skew towards zero. First, it is important to clarify that our method is identifying sites potentially under multilevel selection, while your approach (Bloom & Neher) is quantifying the magnitude of one level of selection. A single parameter is never sufficient to describe a site under multiple levels of selection. Future coalescent-based models we are developing — and will use as input the candidate TFPs given by mlscluster—, will incorporate two selection coefficients (between and within-host) for each site. We believe these findings are the result of the fact that your approach assumes fixed (unchanged) effects. It would, therefore, average out these actual slightly positive and negative multilevel effects because only considering one level of selection. Location of change in manuscript: R1#13 (Methods, Results, and Discussion)
